# Evolution of complex genome architecture in gymnosperms

**DOI:** 10.1093/gigascience/giac078

**Published:** 2022-08-10

**Authors:** Tao Wan, Yanbing Gong, Zhiming Liu, YaDong Zhou, Can Dai, Qingfeng Wang

**Affiliations:** Core Botanical Gardens/Wuhan Botanical Garden, Chinese Academy of Sciences, Wuhan 430074, China; Sino-Africa Joint Research Centre, Chinese Academy of Sciences, Wuhan 430074, China; Key Laboratory of Southern Subtropical Plant Diversity, Fairy Lake Botanical Garden, Shenzhen & Chinese Academy of Science, Shenzhen 518004, China; Department of Ecology, Tibetan Centre for Ecology and Conservation at WHU-TU, State Key Laboratory of Hybrid Rice, College of Life Sciences, Wuhan University, Wuhan 430072, China; Research Center for Ecology, College of Science, Tibet University, Lhasa 850000, China; Key Laboratory of Southern Subtropical Plant Diversity, Fairy Lake Botanical Garden, Shenzhen & Chinese Academy of Science, Shenzhen 518004, China; School of Life Science, Nanchang University, Nanchang 330031, China; School of Resources and Environmental Science, Hubei University, Wuhan, China; Core Botanical Gardens/Wuhan Botanical Garden, Chinese Academy of Sciences, Wuhan 430074, China; Sino-Africa Joint Research Centre, Chinese Academy of Sciences, Wuhan 430074, China

**Keywords:** gymnosperms, genome architecture, genomic shift, diversification

## Abstract

Gymnosperms represent an ancient lineage that diverged from early spermatophytes during the Devonian. The long fossil records and low diversity in living species prove their complex evolutionary history, which included ancient radiations and massive extinctions. Due to their ultra-large genome size, the whole-genome assembly of gymnosperms has only generated in the past 10 years and is now being further expanded into more taxonomic representations. Here, we provide an overview of the publicly available gymnosperm genome resources and discuss their assembly quality and recent findings in large genome architectures. In particular, we describe the genomic features most related to changes affecting the whole genome. We also highlight new realizations relative to repetitive sequence dynamics, paleopolyploidy, and long introns. Based on the results of relevant genomic studies of gymnosperms, we suggest additional efforts should be made toward exploring the genomes of medium-sized (5–15 gigabases) species. Lastly, more comparative analyses among high-quality assemblies are needed to understand the genomic shifts and the early species diversification of seed plants.

## Background

**Figure 1:**
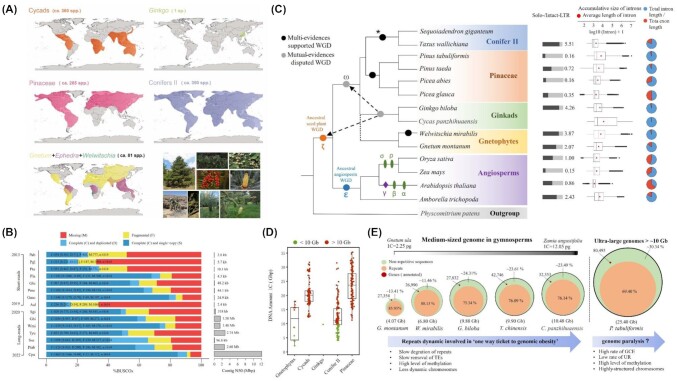
The contemporary overview of the deciphered gymnosperm genomes and the genomic features underpinning their complicated evolutionary history. (A) The geographical distribution of the extant gymnosperms is depicted based on data from the Global Biodiversity Information Facility. The images list the representative gymnosperm species that have been sequenced. (B) Current status of the accumulation of high-quality assemblies of gymnosperms since the advent of long-read sequencing technologies. Abbreviations of the taxa listed from top to bottom: Pab, *Picea abies*; Pgl, *Picea glauca*; Pta, *Pinus taeda*; Pla, *Pinus lambertiana*; Gbi, *Ginkgo biloba*; Pme, *Pseudotsuga menziesii*; Gmo, *Gnetum montanum*; Aal, *Abies alba*; Sgi, *Sequoiadendron giganteum*; Wmi, *Welwitschia mirabilis*; Tyu, *Taxus yunnanensis*; Sse, *Sequoia sempervirens*; Ptab, *Pinus tabuliformis*; Cpa, *Cycas panzhihuaensis*. (C) The prediction and placement of ancient whole-genome duplications (WGDs) in seed plants and the highly contested inference of paleopolyploidy in the most recent common ancestors of all extant gymnosperms. The dashed line indicates the conflicts in the phylogenetic position of gnetophytes. The dashed arrows refer to the controversy on the shared polyploidy event of gymnosperms. The Cupressaceae*-*WGD is highlighted by a “*” since only *Taxus* and *Sequoiadendron* were included (excluding Araucaceae) as representatives of the cupressophytes (left). The available records of the solo-/intact–long terminal repeat (LTR) ratios and the relevance of intron lengths are mapped to each species (right). The data for estimating the solo-/intact-LTR ratios were derived from Nystedt et al. [11], Cossu et al. [52], Wan et al. [24], Cheng et al. [39], Wan et al. [27], and Niu et al. [15]. The data on gene structure were derived from Niu et al. [15]. (D) Genome size distribution across the gymnosperm lineages with medium and ultra-large genome sizes. The 1C-DNA contents were obtained from Niu et al. [15] and the data sources of Kew. (E) The genomic signatures of gymnosperms and the potential genome evolutionary patterns are summarized here with the recent discoveries on recombination and repeat dynamics. TEs, transposable elements; UR, unequal recombination; GCE, gene conversion event.

Over the past 20 years, since *Arabidopsis thaliana* was first sequenced, the number of assembled genomes of seed plants has reached a considerable number (>800) thanks to the fast innovation of sequencing technologies [1, 2]. Among these assemblies, only 2% (17 species, Table 1) are gymnosperms. This is partially attributed to their extraordinarily large genome sizes (>10 Gb on average), complexity [3], and low richness of species [4, 5]. Extant gymnosperms comprise ∼1,100 species encompassing 4 major lineages: cycads, *Ginkgo*, conifers, and gnetophytes (Fig. 1A). Due to the conifers' immense ecological and economic value, great efforts were made to examine the whole genomes of this group [6]. The conifers consist of approximately 615 species covering enormous regions of the Northern Hemisphere and serving as the major backbone of worldwide forest ecosystems [7] (Fig. 1A). A milestone report from early 2013 presented a 23-Gb assembly of loblolly pine (*Pinus taeda*), the first draft genome of a gymnosperm species [8, 9]; a prepublication release of the initial assembly was made in 2012 [10]. Notably, at least 10 conifer genome projects were under way at that time [8]. Another sequencing study on Norway spruce (*Picea abies*) conducted a comparative analysis of the genome architectures of seed plants [11]. Two sets of annotated coding genes (high confidence and low confidence) with a BUSCO ratio <30% indicated there are still considerable gaps and redundancies in this assembly. The small size of the scaffolds (the total length of those with a scaffold size >10 kb is 4.3 Gb) also reflected the objective limits of short-read sequencing, even when using high-coverage Illumina data [11]. Based on samples of the protein-coding and protein-noncoding fractions of the assembly, a plausible model for the conifer genome evolution was proposed: slow rates of activity for a diverse set of retrotransposons and a much lower frequency of recombination in noncoding regions compared to angiosperms [11]. The subsequent investigations revived the scenario of genomic dynamics in conifers, enabling the establishment of giant genomes [12–15] and the study of ecological adaptiveness and phenotypic stasis [16, 17]. With increased data, including transcriptomes and plastid genomes, studies focusing on the phylogenetic relationships among extant gymnosperms triggered great debates regarding various lineages whose studies were based on different data matrices and/or analytical approaches. One of the most controversial issues is the placement of gnetophytes. Several hypotheses have been put forward, suggesting gnetophytes are sisters to Pinaceae (the “Gnepine” hypothesis), cupressophytes (the “Gnecup” hypothesis), all conifers (the “Gnetifer” hypothesis), or all the other gymnosperms [18–22]. The unresolved phylogenetic relationships have encouraged new efforts toward filling in the taxonomic sampling gaps. In the past 5 years, draft maps of *Ginkgo*, gnetophytes, cupressophytes (Conifer II), and cycads have been produced and refined with an improved assembly quality [6, 23–28]. In addition, genome-wide investigations have revealed typical signatures of the gymnosperm genomes, such as ubiquitously large introns and the higher expression levels of long genes [11, 15, 26, 29]. However, the reasons behind the preservation of long genes remain poorly understood.

**Table 1:** List of currently available whole-genome assembly of gymnosperms

Species (common name)	Size of assembly (bp)	Family	Sequencing platform	Online year and relative publication	Link to the assembly data
Pinus taeda* (loblolly pine)	23 G	Pinaceae	Sanger+ Illumina HiSeq 2000	2013 [10]	*^ftp://plantgenie.org/Data/ConGenIE/Pinus_taeda/v1.0/^*
*Picea abies* (Norway spruce)	12.3 G	Pinaceae	Sanger whole-genome shotgun	2013 [11]	*^ftp://plantgenie.org/Data/ConGenIE/Picea_abies/v1.0/^*
*Picea glauca* (genotype PG29) (white spruce)	23.6 G	Pinaceae	Illumina HiSeq 2000, Miseq	2013 [12]	*^ftp://plantgenie.org/Data/ConGenIE/Picea_glauca/PG29/v4.0/^*
*Pinus taeda* (genotype 20–1010) (loblolly pine)	23.2 G	Pinaceae	Illumina GA II, HiSeq 2000, Miseq	2014 [13, 34]	https://treegenesdb.org/FTP/Genomes/Pita/v2.01/
*Picea glauca* (genotype WS77111) (white spruce)	22.4 G	Pinaceae	Illumina HiSeq2500, MiSeq	2015 [16]	*^ftp://plantgenie.org/Data/ConGenIE/Picea_glauca/WS77111/v1.0/^*
*Pinus lambertiana* (sugar pine)	27.6 G	Pinaceae	Illumina GA II, HiSeq 2000/2500, Miseq	2016 [14]	https://treegenesdb.org/FTP/Genomes/Pila/v1.5/
*Ginkgo biloba*	10.6 G	Ginkgoaceae	Illumina Hiseq 2000/4000	2016 [23]	http://gigadb.org/dataset/100209
*Pseudotsuga menziesii* (Douglas fir)	15.7 G	Pinaceae	Illumina HiSeq	2017 [36]	https://treegenesdb.org/FTP/Genomes/Psme/v1.0/
*Gnetum montanum*	4.0 G	Gnetaceae	Illumina HiSeq 2000/2500	2018 [24]	https://doi.org/10.5061/dryad.0vm37
*Abies alba* (silver fir)	18.2 G	Pinaceae	Illumina HiSeq	2019 [37]	https://treegenesdb.org/FTP/Genomes/Abal/v1.1/
*Larix sibirica* (Siberian larch)	12.3 G	Pinaceae	Illumina HiSeq	2019 [35]	https://www.ncbi.nlm.nih.gov/data-hub/genome/GCA_004151065.1/
*Sequoiadendron giganteum* (giant sequoia)	8.1 G	Cupressaceae	Illumina HiSeq + Oxford Nanopore	2020 [38]	https://treegenesdb.org/FTP/Genomes/Segi/v2.0/
*Ginkgo biloba*	9.8 G	Ginkgoaceae	Illumina HiSeq + PacBio RSII	2021 [26]	*^https://ngdc.cncb.ac.cn/bioproject/browse/PRJCA001755^*
*Welwitschia mirabilis*	6.8 G	Welwitschiaceae	Illumina HiSeq + Oxford Nanopore	2021 [27]	https://doi.org/10.5061/dryad.ht76hdrdr
*Taxus chinensis*	10.2 G	Taxaceae	Illumina HiSeq + PacBio RSII	2021 [40]	https://www.ncbi.nlm.nih.gov/data-hub/genome/GCA_019776745.2/
*Taxus wallichiana* (Himalayan yew)	10.9 G	Taxaceae	Illumina HiSeq + Oxford Nanopore	2021 [39]	https://db.cngb.org/search/assembly/CNA0020892/
*Taxus yunnanensis*	10.7 G	Taxaceae	Illumina HiSeq + Oxford Nanopore	2021 [22]	https://www.ncbi.nlm.nih.gov/labs/data-hub/genome/GCA_018340775.1/
*Pinus tabuliformis* (Chinese pine)	25.4 G	Pinaceae	Illumina HiSeq + PacBio RSII	2022 [15]	*https://db.cngb.org/search/project/CNP0001649/*
*Sequoia sempervirens* (coast redwood)	26.5 G	Cupressaceae	Illumina HiSeq + Oxford Nanopore	2022 [6]	https://treegenesdb.org/FTP/Genomes/Sese/v2.1/
*Cycas panzhihuaensis*	10.5 G	Cycadaceae	Illumina HiSeq, Miseq+ Oxford Nanopore	2022 [28]	https://db.cngb.org/codeplot/datasets/public_dataset?id = PwRftGHfPs5qG3gE

* The prepublication release of the assembly was made in 2012 [10]. It contained 18.5 Gbp of sequence with a contig N50 size of 800 bp.

Here, we summarize the progress made in the whole-genome assembly of gymnosperms and describe the considerably varied genomic features observed in different lineages, focusing on the early genome divergence patterns of gymnosperms. We also discuss the concerns relative to inferred paleopolyploid events and provide insights for future research directions. Additionally, we review the current knowledge on the effect of genomic changes on the diversification of gymnosperms and suggest that more efforts should be focused on medium-sized genomes. Finally, to understand the function of long introns, we recommend further examinations with reverse-genetic tools, which can enhance our understanding of plant genome evolution and adaptation.

### The Pulsed Rises in the Whole-Genome Assembly of Gymnosperms

Thus far, compared with flowering plants, the quantities and qualities of the assembled genomes of gymnosperms are relatively lower, with an average BUSCO value of 56.92% computed from 15 decoded species (Fig. 1B). These low values derive from time-consuming projects that were launched several years ago: decades before long-read technologies were developed and became widely used. Also, the species-specific gene sets included in the library may have contributed to the underrepresented annotation of gymnosperms [6]. In terms of high-throughput Illumina sequencing platforms, it often takes 4 to 6 months to obtain clean reads, as a 100× coverage is required for a typical genome of 15 Gb in size and high heterozygosity [30]. Upon the completion of sequencing, the subsequent assembly has further costs, requiring more time and advanced technology. This is because large genomes commonly comprise a variety of repetitive sequences (hereafter called “repeats”), which are untenable with short-read sequencing approaches based on overlapping reads [31, 32]. For example, in the genome project of loblolly pine, although various strategies have been adopted (including fosmid and bacterial artificial chromosome [BAC] clones combined with whole-genome shotgun sequencing [WGS], RNA sequencing, and Bionano sequencing), it was challenging to gain good contiguous contigs, a critical requirement for gene annotation [13]. Additionally, investments in both computational and analytical resources further burdened the progress of genomics research since most assemblers could not handle the incredibly large amount of input sequences from the high-coverage sequencing [33–37].

Thanks to the advanced sequencing technologies of the PacBio RSII and Oxford Nanopore platforms, there has recently been a dramatic increase in the high-quality assembly of these gigantic genomes (Fig. 1B and Table 1). For instance, a refinement of the previous *Ginkgo* draft showed that the contig N50 had remarkably grown from 48 kb to 1.58 Mb in length [23, 26]; also, nearly 95% (9.33 Gb) of the scaffolds had been anchored onto the pseudochromosomes (Fig. 1B). The genomes of 2 iconic species from the Cupressaceae family, the giant sequoia (*Sequoiadendron giganteum*, 8.1 Gb) and the coast redwood (*Sequoia sempervirens*, a hexaploid genome of 26.5 Gb), were successively decoded with conspicuously enhanced contiguity [6, 38]. Additionally, 3 assembly data resources for a single genus, *Taxus*, were released almost simultaneously, reflecting the great interest in the gymnosperm genomes [22, 39, 40]. Notably, all the records provided impressively complete genomes, as suggested by assembly lengths (contig N50 = 2.44 Mb in *Taxus chinensis*, 2.89 Mb in *Taxus yunnanensis*, and 8.60 Mb in *Taxus wallichiana*) and the coverage of the core *Embryophyta* gene library [41] (Fig. 1B). Moreover, the recent sequencing of the haploid megagametophytes of *Cycas panzhihuaensis* showed outstanding assembled quality, with a contig N50 length of 12 Mb [28]. The integrative strategies combining long-read mapping and short-read data polish have been proven possible for almost all species. Also, high-throughput chromosome conformation capture can further assist the sorting of sequences [15, 42].

### Insights into the Repetitive Sequence Dynamics in Gymnosperms

Comparative genomic studies revealed that angiosperm genomes are considerably flexible and dynamic in terms of the rate of DNA sequence integration and elimination [43–45]. Apart from the insertion of viral DNAs, plastids, and mitochondrial sequences, the fluctuation of plant genome sizes is mainly attributed to the historical and ongoing activity of (retro)transposable elements (TEs) (i.e., long terminal repeat retrotransposons [LTR-RTs], which are a major component contributing to the noncoding genomic regions of most seed plant genomes [46–48]). However, many of the angiosperm genomes have a fast turnover of a few million years (Ma) via the proliferation of retrotransposons and unequal recombinations (URs) [49]. Thus, the inevitable genome enlargement was efficiently counteracted by a high rate of DNA excisions [50]. In contrast, the ultra-large (>10 Gb) genomes of gymnosperms are commonly characterized by a relatively low frequency of URs, as evidenced by surveys of the ratio of intact long terminal repeats (LTRs) and solitary LTRs (solo-LTRs) (Fig. 1C). The URs between LTR-RTs often remove the intervening sequences and lead to the formation of solo-LTRs, enabling the ratio of intact versus solo-LTRs to be an indirect proxy for the removal mechanism [51, 52]. The genome-skimming of *P. abies* and *Pinus tabuliformis* identified lopsided numbers of LTRs with much more complete LTRs than solo-LTRs [11, 15]. This is consistent with the patterns observed in other conifers (*P. taeda* and *Picea glauca*) [24, 52]. However, such a signature is atypical in nonconifer gymnosperms, specifically in non-Pinaceae species, regardless of the genome size. Numerous solo-LTRs (60,623) in contrast to much less intact-LTRs (14,128) were detected in the 9.88 Gb of the *Ginkgo* genome [27]. Likewise, a higher ratio of solo- to intact-LTRs (5.5:1) was reported in *T. wallichiana* (10.9 Gb), a species belonging to the cupressophytes [40]. Moreover, 2 gnetophyte species, *Gnetum montanum* (4.13 Gb) and *Welwitschia mirabilis* (6.86 Gb), showed an elevated frequency of the recombination-based removal of retroelements [24, 27]. Hence, the greatly reduced TE elimination activity revealed in Pinaceae might be a family-specific feature generated after their separation from the main conifer clade. Potentially, such kinetic process of TE removal might diverge independently within the lineages, considering the incomplete examination of Pinaceae, especially in those groups of relatively smaller genomes (i.e., the *Larix*). Furthermore, the low occurrence rate of the solo-LTRs in Pinaceae was mostly inferred from either fragmental assembly [11, 52] or the manual examination of randomly sampled contigs/scaffolds [15]. More integrative and genome-wide identifications of these LTRs in high-quality genomes of Pinaceae are needed before we can fully understand the formation of ultra-large genomes. Except for infrequent URs, the reduced activity of other co-occurring processes, such as “illegitimate recombinations,” may also affect the steady growth of genomes in the long term [53]. Mobile elements like LTRs that are repaired by nonhomologous end joining and single-strand annealing may generate truncated or solitary elements, resulting in genome shrinkage [50, 54]. These disarmed LTRs may no longer be autonomous and thus cannot contribute to genome expansion [54]. More data need to be collected concerning the DNA repair by-products of gymnosperms. Also, the comparison between gymnosperms and angiosperms of the proteins and genes (i.e., Ku70/Ku80 [55] and *AtBRCC36A* [56]) involved in such processes is required, especially among those species with distinct genome sizes.

As the prevalent class of TEs, the historical activities of LTRs have a crucial influence on the genome size and the gene structure of plants [57, 58]. All gymnosperms likely share the common feature of repeats’ dynamic as more ancient but continuous amplification of LTRs within a range of 5 to 50 Ma [28, 40]. The estimation of the insertion date is usually determined by the synonymous substitutions per synonymous site (*Ks*) between each 5′-LTR and 3′-LTR flanking sequences, which are calculated based on appropriate mutation rates (per base per year) [59]. The intergenic nucleotide substitution rate of 2.2 × 10^–9^ is normally adopted, assuming that gymnosperms evolved at a slower pace than angiosperms. Thus, the various ages estimated by different studies of the LTR outbreaks of the same gymnosperm could be partially explained by the different neutral mutation rates assigned (i.e., 7.3 × 10^−10^ was used for *T. yunnanensis* and *T. chinensis var. mairei* [22, 40]). It is worth mentioning that the outlier *Welwitschia* has suffered from a very recent expansion of both autonomous and nonautonomous LTRs in less than 1 to 2 Ma, which probably resulted from a cascade of events triggered by intense aridity [27]. The high-resolution categories of retroelements and the use of appropriate mutation rates [60] are both required to distinguish the species-specific expansions that contribute to the diversity in genome growth rhythms [61, 62].

The subsequent ancient insertions and the unusual recent burst of LTRs raise an intriguing question regarding the differences in TE surveillance between gymnosperms and angiosperms since the genome size is generally smaller in the latter. The necessity of TE silencing has been widely acknowledged, and the epigenetic control of DNA sequences is considered the vital nuclear defense system of plant genomes to the destructive potential of TEs [63]. Approaches combining mutations and genome-wide studies of the TE properties in *Arabidopsis* suggested that the Dnmt1-type defense enzyme methyltransferase 1, the plant-specific chromomethylase 3, and the chromatin remodeler decrease in DNA methylation 1 are altogether involved in the DNA methylation of cytosines at CpG and non-CpG loci [64–67].

RNA-directed DNA methylation (RdDM) is an epigenetic pathway that evolved to guide the modeling of DNA condensation and TE silencing [68]. This complicated pathway was first observed in transgenic tobacco infected with viroids, plant pathogens containing solely nonprotein-coding RNA [69]. Despite the limited epigenetic investigations in gymnosperms, several instructive studies provided the general landscape of DNA methylation in the gymnosperm genome [70, 71]. For example, CpG and non-CpG methylations are both surprisingly high in *P. tabuliformis* (88.4% for CG; 81.6% for CHG, the cytosine sequence contexts, H represent A, T or C) and *W. mirabilis* (78.32% for CG; 76.11% for CHG) [15, 27], consistently with previous observations in *P. abies* [72]. Furthermore, global methylation levels positively correlate with genome sizes due to the widespread distribution of TEs along the genome [73, 74]. In addition, the representative genes associated with various methylation pathways have mostly been identified in gymnosperms, implying the probable functional conservation of pathways across seed plants [70]. The activity of RdDMs was further validated by their dynamic changes in the methylation level of specific sequence contexts among different tissue types [27, 70]. The oscillating abundance of 21-nucleotide (nt), 22-nt, and 24-nt Small RNA (sRNAs) indicated that both canonical and noncanonical RdDMs may play a role in TE's control [15, 27], complementing previous hypotheses that 24-nt sRNAs are restricted to the reproductive tissue in *P. abies* [11]. Thus, TE silencing is particularly reinforced by noncanonical RdDMs in gymnosperms, which mildly differs from the primary role of 24-nt RdDMs in angiosperms [15, 72]. However, assessing the extent to which the epigenetic mechanisms contribute to genome methylation and how they contribute to the developmental process is a highly anticipated direction for the genomic studies of gymnosperms. Incidentally, H3K9me, a mark for heterochromatin, showed contrasting distribution patterns between angiosperms and gymnosperms (*P. abies* and *Pinus sylvestris*), implying potential distinctive genome silencing mechanisms [4, 73].

A fundamental shift in repeats’ dynamic has been observed in giant genomes, as indicated by the changes in repeats’ abundance and the curvilinear relationship between genome size and repeats’ proportion among 101 seed plant species (the samples have an approximately 2,400-fold range from 0.063–88.55 Gb in genome size) [74]. In particular, genomes larger than 10 Gb are characterized by the conspicuous increase in nonrepetitive and low-copy DNA sequences (excluding genes) and the relative decrease in medium-copy repeats (>20 copies). Most of these repeats seem to have been slowly degraded and fossilized into very low copy numbers due to epigenetic suppression and limited recombination [74]. In turn, these highly heterogeneous repeats contribute to the formation of interstitial heterochromatin with heavily methylated DNA [57, 75]. Hence, large genomes have “one-way tickets to genomic obesity” [74, 76]. Such genome evolutionary patterns involving derivative retrotransposons may help understand the observation that excess low-repetitive DNA components are overrepresented in the pine genome [61, 77].

### Controversy Regarding Paleopolyploidy and Its Implications for Gymnosperm Diversification

The extant gymnosperms have painted quite a different picture of the rarity of ancient polyploidizations known as whole-genome duplications (WGDs), which are often found with high frequency in flowering plants [20, 78] (Fig. 1C). These events have been suggested as determining factors controlling the lower species abundance in gymnosperms unlike angiosperms [4, 11, 79, 80]. Since postpolyploid diploidization often occurs rapidly and gives rise to many unpredictable consequences, such as chromosome number shifts and DNA loss [81], the inference of ancient WGDs remains highly challenging due to the long-term erosion of genome doubling signals (i.e., loss of duplicates and saturation of synonymous distances [82, 83]).

Combining syntenic analysis with the *Ks* distribution of all paralogous pairs has been vital for distinguishing WGD-derived and small-scale duplication-derived paralogues [84, 85]. However, due to the intermittent release of high-quality genome assemblies of gymnosperms, significant efforts have shifted to comparing genic signatures with improved phylogenomic approaches [20, 78]. Heuristic gene tree–species tree reconciliation methods are broadly employed to search the evidence of ancient WGDs based on transcriptome data [83, 86, 87]. As a result, Li et al. [88] first proposed that there were at least 2 independent WGDs in the ancestry of the major conifer clades (Pinaceae and Cupressaceae) according to the analyses of the transcriptome assemblies of 24 gymnosperms plus 3 outgroup species. This idea was further supported by the distributions of the *Ks* values of syntenic gene pairs among *P. tabuliformis, Sequoiadendron giganteum*, and *Ginkgo biloba* [15]. Furthermore, Li et al. confirmed the seed plant WGD (named *ζ-*) and predicted that a lineage-specific WGD occurred in *Welwitschia*—the latter prediction was validated in a recent *Welwitschia* genome investigation [27]. Another comprehensive study of WGD mapping with a considerably large RNA sequencing sample suggested that a shared WGD might have occurred before all extant gymnosperms diverged [17]. However, such hypothetical WGD cannot be corroborated by most taxonomic-oriented genomic studies [15, 23, 26, 40] (Fig. 1C). Among these genomes, a common feature was the lack of recent species-specific WGDs since only a few intragenomic blocks and syntenic gene pairs could be detected. However, all of the candidate old WGDs hinted by the *Ks* values were accordingly assigned to *ζ-* (i.e., *Ks* = 2.1 in *T. chinensis, Ks* = 1.3 in *P. tabuliformis*, and *Ks* = 0.8 in *G. biloba*). The variable *Ks* values could be attributed to the heterogeneous mutation rate and different versions of phylogenetic analysis by maximum likelihood used. Whereas we fully recognize the salience of the study both for its data sampling and analytical refinement, it still might be vulnerable to the contested phylogenetic relationships remaining in gymnosperms (the placements of *Ginkgo* and gnetophytes) [19–22]. The contentious species-tree topologies probably led to differences in gene duplication mapping, despite the fact that specific nodes were examined [17, 20]. Alternatively, the duplicated genes introduced by the ζ*-*WGD were preferentially retained over the duplicates derived from the gymnosperm-WGD in all the species surveyed. In addition, a *Ks* peak (∼0.8) that was recently observed in the *Cycas* genome was similar to the *Ks* peak of *Ginkgo* [28], suggesting an ancient WGD shared by the 2 lineages as proposed by Roodt et al. [89]. This ancient WGD (named ω-) was further dated to the most recent common ancestors (MRCAs) of all gymnosperms and supported by both transcriptome data and multispecies syntenic block alignments [28]. However, an analysis with a probabilistic approach of the WGD inference against 21 representative seed plants provided clear evidence of the *ζ-*WGD but not of the ω-WGD, rendering the placement of the *Cycas* + *Ginkgo* WGD highly controversial [26, 83] (Fig. 1C).

Given the considerable number of predicted ancient WGDs, based at least on the increased signals of gene duplication (restricted to the WGD-derives) [17, 20], the question was raised regarding how polyploidy contributes to the evolution of gymnosperms. A recent comprehensive measurement of the traits from living and fossil records suggested that 2 ancient pulsed rises of morphological innovation occurred in seed plants’ evolutionary history: the incipient diversification of gymnosperms (ca. 400 Ma) and the subsequent prosperity of angiosperms during the Late Cretaceous (ca. 100 Ma) [90]. The first increase represented by gymnosperms seems to result from the most commonly shared *ζ-*WGD and can be extended to the hypothetical ω-WGD. Two direct correlations between the conifers’ WGD and their diversification shifts [17] likely suggest the potential roles of WGD in the culmination of early gymnosperms (Cupressophyta-WGD and Pinaceae-WGD occurred ca. 200–342 Ma [88]). Besides, considerable evolutionary stasis persisted in the morphological complexity of gymnosperms and was further exacerbated by the emergence of flowering plants [90]. One report linked to a genetic map analysis showed that many more *ζ*-duplicates (688 gene pairs) than conifer-specific tandem duplicates (87 pairs) were preserved in the Pinaceae genomes. A highly conserved genome macrostructure was found between spruce and pine, which diverged at least 120 Ma ago [91]. The large excess of ancestral duplicates and the remarkable level of synteny indicated the much slower pace of evolution in Pinaceae, which can be considered evidence of their relative stasis. Interestingly, a karyotype comparison between Pinaceae and Cupressaceae suggested that substantial chromosomal shuffling likely commenced after their split [92]. Interspecies alignments within the Cupressaceae and other families are required to determine if the shuffling is a common feature of low-frequency genome rearrangements. This would help our understanding of the conifer cladogenesis resulting in speciation and diversity. Moreover, a case of coast redwood (*S. sempervirens*) implied that a very slow diploidization process followed WGD and found the persistence of multisomic inheritance in this hexaploidy species (2n = 66). These findings may contribute to explaining why there are so few polyploid species in modern gymnosperms [92]. Normally, the long-term benefits of polyploidy require the divergence among homologous chromosomes, which can only happen once loci are diploidized [81, 93]. In turn, the reduced selection of efficient meiosis in *Sequoia* would preclude the emergence of any evolutionary advantages in polyploidy lineages. Hence, Scott et al. [93] proposed that such an intriguing evolutionary strategy was additionally reinforced by asexual reproduction, self-compatibility, and extreme longevity, which likely took place in other conifers, such as *Fizroya cupressoides* [94]. Aside from this, the fundamental dynamic shift in repeats is noteworthy, assuming that the genomic shift occurred early in gymnosperms, probably before most modern lineages diverged. The ancestral genome size of gymnosperms has been estimated to have been ∼12.375 to 15.75 Gb [95]. If so, heterogeneous rates of genome size evolution should be expected considering the large range in 1C-DNA content (i.e., from 2.21 Gb in *Gnetum ula* to 35.28 Gb in *Pinus ayacahuite*) exhibited across gymnosperms [15] (Fig. 1D and E). The shift in genomic dynamics could directly lead to the unfavorable architecture of those large genomes as constrained chromosomal homogenization. Together with the slow pace of diploidization, these factors make polyploidy a burden rather than a boon in gymnosperms. Therefore, the extraordinarily massive loss of duplicates should not surprise due to the highly structured chromosomes and severely limited recombination of these genomes [4]; hence, most signals of WGD in the doubled genome were expunged (e.g., to date, *W. mirabilis* is the only gymnosperm species known to have a family-specific WGD that occurred ∼86 Ma ago while showing an extremely low level of intrachromosomal syntenic relationships compared to angiosperms) [27]. The unusually low rate of WGD duplicate retention could further restrain the morphological and biological diversity of these lineages, given that polyploidy often introduces sub- or neofunctionalization and increases variations in dosage-sensitive genes and pathways [96–98]. To conclude, the concomitant problems imposed by an enlarged genome could affect the diverse physiological processes of plants, such as longer cell cycles [99, 100] and higher nutrient costs [4], which eventually impact the competitiveness of the species.

### Intriguing Intron Morphology and Evolution in Gymnosperms

The presence of astonishingly long genes has been extensively reported in many gymnosperms from distinct lineages [11, 15, 23] (Fig. 1C). These long genes are often associated with large amounts of intronic sequences characterized by cumulative size distributions, including numerous atypical long ones (>20 kb) [11, 15, 23, 28]. Why these very long introns are preserved and how they influence the evolution and function of genes in gymnosperms remain largely obscure [15].

It has long been acknowledged that the genome size may be correlated with the intron size across broad phylogenetic groups. However, such a pattern was poorly translated into some narrow taxonomic distant groups of angiosperms [101]. A pioneering description and comparison of the gene structures of *P. glauca* and *P. taeda* with data from BAC clones and genome scaffolds indicated a relatively conserved signature in the long introns [29]. Moreover, the high frequency (32%) of the TEs found in captured sequences, even in introns <1 kb, suggested the important role of such invasive elements in the long gene space [29]. Niu et al. [15] tabulated the characteristics of the gene structures among 68 recently sequenced seed plants. They found a positive correlation between the ratio of total intron/exon length and the genome size, especially in gymnosperm lineages (Fig. 1C). Collectively, this robust evidence supports the claim that genic expansion was coupled with the genome upsizing in the majority of gymnosperms, which is probably attributed to the slow growth and accumulation of repeats [15]. Additionally, Nystedt et al. [11] first provided insights into the presence of long introns by comparing the orthologues of the normal-sized (50–300 bp) and long (1–20 kb) introns of *P. abies, P. sylvestris*, and *G. montanum*. They suggested that an early intron expansion might have already occurred in the MRCAs of all conifers, which would explain the identical trend in the increased length of orthologous introns. However, this point of view was changed by subsequent comparisons conducted within more species of early diverged seed plants [24]. Similar growth patterns of the intron size and content were observed in orthologues between *Ginkgo* and *P. taeda* with the accumulation of LTR-RTs (especially Ty1-*copia* elements). By contrast, a high proportion of long interspersed nuclear elements (LINEs) was found in orthologous long introns between *G. montanum* and *Amborella trichopoda* (the “basal” angiosperm [102]), and both these species involved the expansion of long introns, consistently with the scenario of all intron morphology in *G. montanum* and *A. trichopoda* [24]. This result might indicate different repeat dynamics within the introns of *G. montanum* compared with other gymnosperms, and the level of Ty1-*copia* activity in introns might be more ancient and could be traced back to the origin of gymnosperms. Likewise, LINEs could be partially involved in the intron evolution of ancestral seed plants [24]. However, these hypotheses require more investigations using closely related or representative species like *Welwitschia, Ephedra*, and even *Cycads*, because the evolution of the gene structure of plants was determined by many more interacting forces than classically expected (i.e., the selective recombination rate [103, 104] and the species-specific TE activity [105, 106]). Indeed, a large portion of unknown sequences has been found in *Cycas*’ introns, which is quite different from the pattern of LTR or LINE dominance found in other gymnosperms [28].

Exploring the biological relevance of long introns could be insightful for addressing a fundamental scientific inquiry: “Why are some genomes really big and others quite compact?” Unfortunately, this matter has been poorly addressed in gymnosperms [29] except for a very recent description of gene expression profiles, alternative splicing, and DNA methylation [15]. The atypically long introns seem to have minimal influence on transcript accuracy, probably facilitated by different levels of CpG and non-CpG methylations among exons and introns [15]. These results call for similar examinations in other giant gymnosperm genomes, such as *Ginkgo* or *Welwitschia*, considering their lower effective population size compared to conifers since the loosening of natural selection often allows the fixation of potentially deleterious mutations in the genome [107]. In addition, long genes tend to have higher expression levels in *P. tabuliformis*, similar to the situation observed in *P. glauca, Oryza sativa*, and *A. thaliana* [29, 108]. However, such a pattern contrasts with other organisms, like *Physcomitrium patens* [109], *Caenorhabditis elegans*, and *Homo sapiens* [110], where compact genes are highly expressed. If so, the “low-cost transcription hypothesis” is probably unsuitable for gymnosperms. Alternatively, the length of introns is likely less relevant to the expression level since introns are involved in a variety of regulatory phenomena (i.e., posttranscriptional gene regulation [111], nucleosome formation, and chromatin organization [112–114]). Nevertheless, the correlation between gene length and gene expression should be interpreted with caution and is likely caused by technical issues: the statistical bias in RNA sequencing data due, for instance, to the overcount reads from long transcripts [102].

## Conclusion and Perspectives

In this review, while appreciating the advances in our knowledge of the genome evolution of gymnosperms, we demonstrated that some essential characteristics, such as repeat dynamics, ancient WGD inference, and the biological relevance of long introns, are far from understood. The state of “genome paralysis” may be confined to Pinaceae rather than all conifers or gymnosperms since a high frequency of TE removal does exist in cupressophytes, gnetophytes, and *Ginkgo*. The hypothetical ω-WGD is still highly contested and needs to be reconsidered by future studies. The sporadic and long-awaited releases of genome drafts inevitably limit the conclusions of species-specific cases. Despite the low level of cladogenesis and the rarity of polyploids, the fundamental shift of genomic dynamics and the potential signature of the slow process of diploidization probably offer new insights into the complex evolution of the genome architectures of gymnosperms. Additionally, the dominant model of recent allopolyploidy speciation in *Ephedra* [115], as well as the growing number of species on the list of hybridization and polyploidization in *Juniperus* [116], contrasts with the gymnosperm reputation of being composed of ancient species. These results could be explained by the resurgence of gymnosperm diversification and the increase in habitat ranges [17]. With regards to all these aspects, we envisage that gymnosperms could be a candidate model to investigate the changes in genome dynamics and their influence on species diversifications (Fig. 1E). However, in-depth studies on the wealth of information contained within these genomes cannot be conducted without generating more high-quality assemblies. The investigation of interspecific variations and diverse properties in gymnosperms would be more profound if the data sampled were consistent, as in many excellent works conducted on animals or crops [117, 118]. Considering the intricate evolutionary history of gymnosperms, we propose that, in the future, attention should be paid to at least the 4 aspects next described. First, more integrative estimations of TE eliminations are needed, and a high-resolution subclassification of the TEs would help to distinguish family-specific expansion patterns. Intensive studies on the many repetitive relics with a low copy number would also enable us to illustrate the formation of the highly structured and less dynamic chromosomes of gymnosperms [4, 11, 75]. Finally, the rapid accumulation of epigenetic data is imperative since variable repeat dynamics and sophisticated epigenetic machinery play crucial roles in gymnosperms. These data should be either at the single-base resolution of DNA methylation or for comparing methylomes among different tissues. Second, ancestral paleopolyploidy inferences should be investigated by large-scale multialignments of more complete gymnosperm assemblies with fully considered phylogenies. In particular, the structural evidence of intra- and interspecies collinearity may be essential to clarify the number and timing of these ancient duplications [82]. Moreover, the comprehensive evaluation of the loss and retention of duplicate genes could help elucidate the potential heterogeneity in the genome evolution of gymnosperms. Third, it may be worthwhile to include intron length and expression characteristics in future whole-genome studies of gymnosperms. Also, more investigations on alternative splicing patterns should be carried out and analyzed together with DNA methylation footprints. Despite the lack of appropriate genetic transformation tools for long-lived perennial species, it might be insightful to conduct analogous molecular experiments in model plant systems concerning the potential biological functions of ultra-long genes [15, 119]. Finally, more chromosome-level genomes of gymnosperms are needed. However, we suggest that additional efforts should be made to sequence medium-sized (5–15 G) species and refine the short-read drafts released for conifers, especially Pinaceae.

## Data Availability

BAC (bacterial artificial chromosome); BUSCO (Benchmarking Universal Single-Copy Orthologs); CMT3 (chromomethylase 3); DDM1 (Decrease in DNA Methylation 1); GCE (gene conversion event); Hi-C (highthroughput chromosome conformation capture ); Ks (the synonymous substitutions per synonymous site); LINEs (long interspersed nuclear elements); LTR-RTs (long terminal repeat retrotransposons); Ma (million years); MET1 (Dnmt1-type defence enzyme methyltransferase); MRCA (the most recent common ancestors); RdDM (RNA-directed DNA methylation); TEs ((retro)transposable elements); UR (unequal recombination); WGDs (whole-genome duplications); WGS (whole-genome shotgun sequencing).

## Competing Interests

The authors declare no competing interests.

## Funding

This work was supported by the Scientific Research Program of Sino-Africa Joint Research Center (grant no. SAJC202105) and the National Natural Science Foundation of China grants (grant no. 31 870 206).

## Authors' Contributions

T.W. and Q.F.W. designed the outline of the manuscript. T.W. and Y.B.G. wrote the manuscript. T.W., C.D., and Q.F.W. polished the article. Z.M.L. and Y.D.Z. worked on the revisitation of the genomic data. T.W. and Y.B.G. are joint first authors.

## Supplementary Material

giac078_Authors_Response_To_Reviewer_Comments

giac078_GIGA-D-22-00123_Original_Submission

giac078_GIGA-D-22-00123_Revision_1

giac078_GIGA-D-22-00123_Revision_2

giac078_Response_to_Reviewer_Comments_Original_Submission

giac078_Reviewer_1_Report_Original_Submission Steven L. Salzberg, Ph.D. -- 1/15/2022 Reviewed

giac078_Reviewer_1_Report_Revision_1 Steven L. Salzberg, Ph.D. -- 6/21/2022 Reviewed

giac078_Reviewer_2_Report_Original_Submission David Neale -- 6/3/2022 Reviewed
