# Supplementary material for: Evolution of complex genome architecture in gymnosperms
Source: Gigascience. 2022 Aug 10;11:giac078. doi: 10.1093/gigascience/giac078 (PMC9364684; doi:10.1093/gigascience/giac078)

GigaScience

Complexity of giant genome evolution in gymnosperms

--Manuscript Draft--

**Manuscript Number:**	GIGA-D-22-00123
**Full Title:**	Complexity of giant genome evolution in gymnosperms
**Article Type:**	Review
**Funding Information:**	
**Abstract:**	Gymnosperms represent an ancient lineage that diverged from early spermatophytes during the Devonian. The long fossil records and low diversity in living species indicate their complex evolutionary history including ancient radiation and massive extinctions. Limited to giant genome size with abundant repetitive sequences, the whole genome assembly of gymnosperms has only sprung up in the past ten years and further expanded into more taxonomic representations. Here, we provide a contemporary view of publicly available gymnosperm genomic resources, including assembly quality and large genome evolution advances. We present our current understanding of these progresses while proposing revisitations of more high-quality assemblies. Based on the results of extensive genomic studies, we highlight gymnosperms as candidate models for inquiry into genomic shifts and early species diversification in seed plants.
**Corresponding Author:**	Tao Wan<br>Wuhan Botanical Garden<br>Wuhan, CHINA
**Corresponding Author Secondary Information:**	
**Corresponding Author's Institution:**	Wuhan Botanical Garden
**Corresponding Author's Secondary Institution:**	
**First Author:**	Tao Wan
**First Author Secondary Information:**	
**Order of Authors:**	Tao Wan
**Order of Authors Secondary Information:**	
**Additional Information:**	
**Question**	**Response**
Are you submitting this manuscript to a special series or article collection?	No
**Experimental design and statistics**<br><br>Full details of the experimental design and statistical methods used should be given in the Methods section, as detailed in our Minimum Standards Reporting Checklist. Information essential to interpreting the data presented should be made available in the figure legends.<br><br>Have you included all the information requested in your manuscript?	Yes

Powered by Editorial Manager® and ProduXion Manager® from Aries Systems Corporation

**Resources**<br><br>A description of all resources used, including antibodies, cell lines, animals and software tools, with enough information to allow them to be uniquely identified, should be included in the Methods section. Authors are strongly encouraged to cite Research Resource Identifiers (RRIDs) for antibodies, model organisms and tools, where possible.<br><br>Have you included the information requested as detailed in our Minimum Standards Reporting Checklist?	No
If not, please give reasons for any omissions below.<br><br>as follow-up to "**Resources**<br><br>A description of all resources used, including antibodies, cell lines, animals and software tools, with enough information to allow them to be uniquely identified, should be included in the Methods section. Authors are strongly encouraged to cite Research Resource Identifiers (RRIDs) for antibodies, model organisms and tools, where possible.<br><br>Have you included the information requested as detailed in our Minimum Standards Reporting Checklist?<br><br>"	N/A
**Availability of data and materials**<br><br>All datasets and code on which the conclusions of the paper rely must be either included in your submission or deposited in publicly available repositories (where available and ethically appropriate), referencing such data using	No

Powered by Editorial Manager® and ProduXion Manager® from Aries Systems Corporation

a unique identifier in the references and in the “Availability of Data and Materials” section of your manuscript.<br><br>Have you have met the above requirement as detailed in our Minimum Standards Reporting Checklist?	
If not, please give reasons for any omissions below.<br><br>as follow-up to "**Availability of data and materials**<br><br>All datasets and code on which the conclusions of the paper rely must be either included in your submission or deposited in publicly available repositories (where available and ethically appropriate), referencing such data using a unique identifier in the references and in the “Availability of Data and Materials” section of your manuscript.<br><br>Have you have met the above requirement as detailed in our Minimum Standards Reporting Checklist?<br><br>"	N/A

Review Article

Complexity of giant genome evolution in gymnosperms

Tao Wan[1,2,3], Yanbing Gong[4,5], Zhiming Liu[3], YaDong Zhou[6], Can Dai[7], Qingfeng Wang[1,2]*

[1]Core Botanical Gardens/Wuhan Botanical Garden, Chinese Academy of Sciences, Wuhan, China.

[2]Sino-Africa Joint Research Centre, Chinese Academy of Sciences, Wuhan, China

[3]Key Laboratory of Southern Subtropical Plant Diversity, Fairy Lake Botanical Garden, Shenzhen & Chinese Academy of Science, Shenzhen, China

[4]Department of Ecology, Tibetan Centre for Ecology and Conservation at WHU-TU, State Key Laboratory of Hybrid Rice, College of Life Sciences, Wuhan University, Wuhan, China

[5]Research Center for Ecology, College of Science, Tibet University, Lhasa, China

[6]School of Life Science, Nanchang University, Nanchang 330031, China

[7]School of Resources and Environmental Science, Hubei University, Wuhan, China

Abstract

Gymnosperms represent an ancient lineage that diverged from early spermatophytes during the Devonian. The long fossil records and low diversity in living species indicate their complex evolutionary history including ancient radiation and massive extinctions. Limited to giant genome size with abundant repetitive sequences, the whole genome assembly of gymnosperms has only sprung up in the past ten years and further expanded into more taxonomic representations. Here, we provide a contemporary view of publicly available gymnosperm genomic resources, including assembly quality and large genome evolution advances. We present our current understanding of these progresses while proposing revisitations of more high-quality assemblies. Based on the results of extensive genomic studies, we highlight gymnosperms as candidate models for inquiry into genomic shifts and early species diversification in seed plants.

Key words: gymnosperms, large genome evolution, genomic shift, diversification

Background

With the accelerated innovation in sequencing technologies, the number of assembled genomes of seed plants has reached a considerable number (> 800) over the past 20 years since *Arabidopsis thaliana* was first sequenced [1,2]. Among these assemblies, only 2% (17 species, Table 1) are for gymnosperms, which is partially attributed to their extraordinarily large genome sizes (>10 gigabases (Gb) on average) and complexity [3], as well as their lower species richness [4,5]. The modern gymnosperms comprise ~1,100 species encompassing four major lineages: cycads, *Ginkgo*, conifers, and *gnetophytes* (**Fig. 1A**). Due to conifers' immense ecological and economic value, great efforts to examine the whole genome have been focused within this group [6], a phylum consisting of approximately 615 species covering enormous regions of the Northern Hemisphere and serving as major backbones of worldwide forest ecosystems [7] (**Fig. 1A**). A milestone report in 2013 presented a 20-Gb genome of Norway Spruce (*Picea abies*) and comparative analyses of its genome architecture with other seed plants

[8]. Two sets of annotated coding genes (high-confidence and low-confidence) with a BUSCO (Benchmarking Universal Single-Copy Orthologs) ratio of less than 30% indicated considerable gaps and redundancy within the genome. The small size of the scaffolds (4.3 Gb with length > 10 kb) also reflected the objective limits of short-read sequencing, even when using high-coverage Illumina data [8]. Based on sampling of the protein-coding and noncoding fractions of the assembly, a probable model for conifer genome evolution was proposed: slow rates of activity for a diverse set of retrotransposons, coupled with a much lower frequency of recombination in noncoding regions compared to angiosperms [8]. Continuous investigations have revived the scenario of genomic dynamics in conifers, which enabled the establishment of a giant genome [9-12], as well as ecological adaptiveness and phenotypic stasis [13,14]. With the increase in data, including transcriptome and plastid genomes, episodes focusing on phylogenetic relationships among extant gymnosperms have triggered great debates across lineages based on different data matrices and/or analytical approaches [15-19]. One of the most controversial questions is the placement of *gnetophytes*, and several possible hypotheses have been put forward, suggesting *gnetophytes* as sister to *Pinaceae* ('Gnepine' hypothesis), to *Cupressophytes* ('Gnecup' hypothesis), to all conifers ('Gnetifer' hypothesis), or to all other gymnosperms. The unresolved phylogenetic relationships have encouraged strivings to fill taxonomic sampling gaps; thus, in the last five years, draft maps of *Ginkgo*, *gnetophytes*, *Cupressophytes* (Conifer II) and *Cycads* have been produced and greatly refined with improved assembly quality [6, 21-26]. In addition, genome-wide investigation has revealed the typical signature of gymnosperms as ubiquitously large size of introns and higher expression levels of long genes [8,12,23,26]. However, the reasons for the preservation of long genes in gymnosperms remain poorly understood.

Here, we summarize the whole genome assembly progress in gymnosperms with the advent of short- and long-read sequencing. Then, we describe the considerable variation in genomic features observed in different lineages and discuss the predictions of early genome divergence patterns in gymnosperms. We also dissect the inferred paleopolyploid events and provide insights for future research directions. We overview the current knowledge on the effect of genomic change on the diversification of gymnosperms and suggest that more efforts be focused on medium-sized genomes in subsequent studies. Finally, to understand the function of long introns, we recommend further tests and advances that can enhance our understanding of plant genome evolution and adaptations.

Pulsed rises in whole genome assembly of gymnosperms

To date, compared with flowering plants, the quantities and qualities of assembled genomes for gymnosperms are relatively lower, with BUSCO values of 56.92 %, which are averages of 15 decoded species (**Fig. 1B**). These lower values are from time-consuming projects that were launched several years ago, decades before long-read technology arose and was widely applied.

For example, ‘The Loblolly Pine Genome Project’ was probably initiated in 1995, but data from this project was not publicly available until 2014 with rounds of sequencing data supply [10]. In terms of high-throughput Illumina sequencing platforms, it often takes 4~6 months to obtain clean reads as 100× coverage is required for a typical genome with size of 15 Gb and high heterozygosity [27]. Upon the completion of sequencing, subsequent assembly also requires more time, cost and advanced technology because large genomes commonly comprise a variety of repetitive sequences (hereafter repeats), which are untenable with short-read sequencing approaches based on overlapping reads [28,29]. Although various strategies have been adopted, including Fosmid or BAC (Bacterial Artificial Chromosome) clones combined with whole genome shotgun sequencing (WGS), RNA-seq, and Bionano-seq, it remains challenging to gain better contiguous contigs, which is critical for gene annotation [10,30,31]. Beyond that, investments in both computational and analytical resources further burdened the march on genomics research since most assemblers could not handle the incredibly large amount of input sequences from the high coverage sequencing [32]. Similar tough jobs underwent in the other conifer species including Douglas-fir (*Pseudotsuga menziesii*) [33] and silver fir (*Abies alba*) [34].

Thanks to the progressive sequencing technologies represented by the PacBio RSII and Oxford Nanopore platforms, there has been a dramatic recent increase in the high-quality assembly of these gigantic genomes (**Fig. 1B** and **Table 1**). For example, the refinement of previous *Ginkgo* draft release showed that the contig N50 had been remarkably improved from a length of 48 kb to 1.58 Mb [20,23], and nearly 95% (9.33 Gb) of the scaffolds had been anchored onto the pseudochromosomes (**Fig. 1B**). Two genomes of iconic species from Cupressaceae family, giant sequoia (*Sequoiadendron giganteum*, 8.1 Gb) and coast redwood (*Sequoia sempervirens*, hexaploid genome of 26.5 Gb), were successively decoded with conspicuous enhanced contiguity [6,35]. Beyond that, three assembled resources for a single lineage, *Taxus*, were released almost simultaneously, which reflected the great interest in the gymnosperm genome [19,36,37]. Notably, all of these studies displayed impressive completeness of the predicted genome, as indicated by both assembly length (contig N50 = 2.44 Mb in *Taxus chinensis*; 2.89 Mb in *T. yunnanensis*; 8.60 Mb in *T. wallichiana*) and coverage in the core *Embryophyta* gene library [38] (**Fig. 1B**). Moreover, a recent sequencing record of haploid megagametophytes of *Cycas panzhihuaensis* showed outstanding assembled quality, with a contig N50 length of 12 Mb [25]. The integrative strategies combined with long-read mapping and short-read data polish have been shown to be possible for almost all species, and high-throughput chromosome conformation capture (Hi-C) can further assist in the sorting of sequences [12,39].

Insights into repetitive sequence dynamics in gymnosperms

Comparative genomic studies have revealed that angiosperm genomes are considerably flexible

and dynamic in terms of the rate of DNA sequence integration and elimination [40-42]. Apart from the insertion of viral DNA, plastid and mitochondrial sequences, the fluctuation in plant genome size is mainly attributed to the historical and ongoing activity of (retro)transposable elements (TEs) (i.e., long terminal repeat retrotransposons (LTR-RTs), which a major component contributing to the noncoding genomic regions in most seed plant genomes [43-45]). However, many of the angiosperm genomes have fast turned over within a few million years (Ma) via retrotransposon proliferation and unequal recombination (UR) [46]. Thus, the inevitable genome enlargement was efficiently counteracted by the related high rate of DNA excision [47]. In contrast, the ultra-large (>10 Gb) genomes of gymnosperms are commonly associated with a relatively low frequency of UR, as evidenced by surveys of the ratio of intact LTRs and solitary LTRs (solo-LTRs) (**Fig. 1C**). The UR between LTRs often leads to the removal of intervening sequences and the formation of solo-LTRs, which enables the ratio of intact to solo-LTRs as an indirect proxy for the removal mechanism [48,49]. Genome-skimming in *P. abies* and *Pinus tabuliformis* identified lopsided numbers of LTRs, with much more complete LTRs than solo-LTRs [8,12]. Similarly, the consistent patterns were revealed in other conifers (*P. taeda* and *P. glauca*) based on patrol of fragmented assemblies [21,49]. However, such a signature is atypical in non-conifer gymnosperms, more precisely, non-*Pinaceae* species, regardless of genome size. Numerous solo-LTRs (60,623) in contrast to much less intact LTRs (14,128) were detected in the 9.88 Gb *Ginkgo* genome [24]. Likewise, higher ratio of solo-LTRs to intact-LTRs (5.5:1) was reported in *T. wallichiana* (10.9 Gb), a species belonging to *Cupressophytes* [37]. Moreover, two species in *gnetophytes*, *Gnetum montanum* (4.13 Gb) and *Welwitschia mirabilis* (6.86 Gb), both showed elevated frequency of recombination-based removal of retroelements [21,24]. Hence, the greatly reduced TE elimination activity observed in *Pinaceae* might be a family-specific feature derived after their separation from the main conifer clade. Alternatively, such kinetic process of TE removal diverged independently within lineages, and ‘genome paralysis’ was triggered once the genome obesity threshold was reached [8]. Additionally, the low rate of occurrence of solo-LTRs in *Pinaceae* was mostly inferred from either incomplete assemblies [8,49] or manual examination of randomly sampled contigs/scaffolds [12]. More integrative and genome-wide identification of these LTRs in high-quality genomes of *Pinaceae* is primarily needed before we can fully understand the formation of such super giant genomes. Except for infrequent UR, reduced activity of other cooccurring processes, such as ‘illegitimate recombination’, might also affect the progressive growth of the genome in the long term [50]. Mobile elements like LTRs that are repaired by non-homologous end joining (NHEJ) and single-strand annealing will generate truncated or solitary elements, resulting in genome shrinkage [47,51]. These disarmed LTRs may no longer be autonomous and thus cannot contribute to genome expansion as a whole [51]. More data needs to be collected on the spectrum of DNA repair by-products in gymnosperms. The comparison of proteins and genes (i.e., Ku70/Ku80 [52] and *AtBRCC36A* [53]) involved in such process is

also required between gymnosperms and angiosperms, especially among those species with distinct genome size.

As the prevalent class of TEs, the historical activities of LTR have crucial influences on genome size and gene structure in plants [54,55]. All gymnosperms likely share a common feature of repeats dynamic as more ancient but continuous amplification and insertion of LTRs within a range of 5-50 Ma [25,37]. The estimation of insertion date is usually determined by the synonymous substitutions per synonymous site (*Ks*) between each 5′-LTR and 3′- LTR flanking sequences, which are further calculated based on appropriate mutation rates (per base per year) [56]. The intergenic nucleotide substitution rate of 2.2×10^{-9} is normally adopted, assuming that gymnosperms evolved at a slower pace than angiosperms. Thus, the various ages of LTR outbreaks from different studies of the same gymnosperm observed were partially explained by the different neutral mutation rates assigned (i.e., 7.3×10^{-10} used in *T. yunnanensis* and *T. chinensis var. mairei* [19,37]). It is worth mentioning that the outlier *Welwitschia* has suffered from a very recent expansion of both autonomous and nonautonomous LTRs in less than 1-2 Ma, which probably results from a cascade event triggered by intense aridity [24]. The high-resolution identification of retroelements and the use of appropriate mutation rates [57] are both required to distinguish species-specific expansions, which contribute to the diversity in genome growth rhythm [58,59].

Nevertheless, both successive ancient insertions and the unusual very recent burst of LTRs raise the intriguing question regarding the differences in TE surveillance between gymnosperms and angiosperms since the size of the genome is generally smaller in the latter. The necessity of TE silencing has been widely acknowledged, and epigenetic control of DNA sequences is considered the vital nuclear defence system in plant genome to the destructive potential of TEs [60]. Approaches combining mutations and genome-wide studies of TE properties in *Arabidopsis* suggested that Dnmt1-ty defense pe enzyme methyltransferase (MET1), plant-specific chromomethylase 3 (CMT3) and the chromatin remodeler DECREASED DNA METHYLATION 1(DDM1) are altogether greatly involved in the DNA methylation of cytosine at CpG and non-CpG loci [61-64]. Moreover, RNA-directed DNA methylation (RdDM) was introduced to guide the modelling of DNA condensation and TE silencing [65], and this complicated epigenetic pathway was first observed in transgenic tobacco infected with viroids, which are plant pathogens containing solely nonprotein-coding RNA [66]. Despite limited epigenetic investigations in gymnosperms, several instructive studies have provided the general landscape of DNA methylation in gymnospermous genome [67,68]. For example, the CpG and non-CpG methylation levels are both incredibly high in *P. tabuliformis* (88.4% for CG; 81.6% for CHG) and *W. mirabilis* (78.32% for CG; 76.11% for CHG) [12,24], which is consistent with previous observations in *P. abies* [69]. Global methylation levels are positively correlated with genome size due to the widespread distribution of TEs along genomes [70,71]. In addition, the representative genes associated with various methylation pathways have been mostly identified

in gymnosperms, which implies the probable functional conservation of pathways across seed plants [67]. The activities of RdDM were further evidenced by dynamic changes in the methylation level of specific sequence contexts among different tissue types [24,67]. The oscillating abundance of 21 nt, 22 nt, and 24 nt sRNAs indicated that both canonical and noncanonical RdDMs might play a role in TE control [12,24], complementing previous hypotheses that 24 nt sRNAs are restricted to reproductive tissue in *P. abies* [8]. Thus, TE silencing is particularly reinforced by noncanonical RdDMs in gymnosperms, which mildly differs from the primary role of 24 nt-RdDMs in angiosperms [12,69]. However, assessment of the extent to which epigenetic mechanisms contribute to genome methylation and how they function in the developmental process is still a very anticipated direction for genomic studies of gymnosperms. At least, a mark for heterochromatin, H3K9me, showed contrasting distribution patterns between angiosperms and gymnosperms (*P. abies* and *P. sylvestris*), implying potential distinctive genome silencing mechanisms [4,70].

Besides, a fundamental shift of repeats dynamic has been observed in giant genomes, as indicated by changes in repeats abundance and the curvilinear relationship between genome size and repeats proportion among 101 seed plant species (an approximately 2,400-fold range from 0.063-88.55 Gb in genome size) [71]. In detail, genomes larger than 10 Gb are characterized by a conspicuous increase in nonrepetitive and low-copy DNA sequences (excluding genes) but a relative decrease in medium-copy repeats (> 20 copies). A majority of these repeats seem to have been slowly degraded and fossilized into very low copies due to epigenetic suppression and limited recombination [71]. In turn, these highly heterogenous repeats contribute to the formation of interstitial heterochromatin with heavily methylated DNA [54,72]. Inevitably, the genomes become 'trapped' in mode as a "one way ticket to genomic obesity" [71,73]. This genome evolutionary pattern involving derivative retrotransposons is in accordance with the previous characterization of excess low-repetitive DNA components being overrepresented in the pine genome [58,74].

Controversy regarding paleopolyploidy and implications for gymnosperm diversification

Extant gymnosperms have painted a quite different picture with rarity in ancient polyploidization known as whole genome duplications (WGDs), which are often found in high frequency in flowering plants [17,75] (**Fig. 1C**). These events have been suggested as determining factors controlling the poorer species richness in gymnosperms compared to the astonishing rapid diversification in angiosperms [4,8,76,77]. Because postpolyploid diploidization often occurs rapidly and gives rise to many unpredictable consequences, such as chromosome number shifts and DNA loss [78], the inference of ancient WGDs remains highly challenging due to the long-term erosion of genome doubling signals (i.e., duplicates loss and saturation of the synonymous distances [79,80]).

The combination of syntenic and *Ks* information has been vital for the discrimination of WGD-derived and small scale duplication-derived paralogues [81,82]. However, limited by the intermittent release of high-quality genome assemblies of gymnosperms, great efforts have shifted to the comparison of genic signatures with improved phylogenomic approaches [17,75]. Heuristic gene tree–species tree reconciliation methods are broadly employed to search the evidence of ancient WGDs based on transcriptome data [80,83,84]. Benefiting from this, Li et al. (2015) [85] first proposed that there were at least two independent WGDs in the ancestry of major conifer clades (*Pinaceae* and *Cupressaceae*) in analyses of the transcriptome assemblies of 24 gymnosperms plus three outgroup species. This idea was further supported by the distributions of *Ks* values for syntenic gene pairs among *P. tabuliformis*, *Sequoiadendron giganteum*, and *G. biloba* [12]. Furthermore, Li *et al.* confirmed the seed plant WGD (named ζ-) and predicted that a lineage-specific WGD occurred in *Welwitschia*, the latter of which was validated in a recent *Welwitschia* genome investigation [24]. Another comprehensive study of WGD mapping with a considerably large RNA-seq sample suggested that a shared WGD might have occurred before all extant gymnosperms diverged [14]. However, such hypothetical WGD cannot be corroborated with most taxonomic-oriented genomic studies [12,20,23,37] (**Fig. 1C**). Among these genomes, a common feature was the lack of recent species-specific WGDs since only a few intragenomic blocks and syntenic gene pairs could be detected. However, all of the candidate older WGDs hinted by *Ks* value were accordantly assigned to ζ- (i.e., *Ks*=2.1 in *Taxus chinensis*, *Ks*=1.3 in *P. tabuliformis*, and *Ks*=0.8 in *G. biloba*). The variable *Ks* values could be attributed to the heterogeneous mutation rate and different versions of PAML used. With full respect to the salience of above study both in data sampling and analytical refinement, it still might be vulnerable to the contested phylogenetic relationships remaining in gymnosperms as a whole (the placements of *Ginkg*o and *gnetophytes*) [16-19]. The contentious species-tree topologies probably lead to differences in gene duplication mapping despite specific nodes being examined [14,17]. Alternatively, the duplicated genes introduced by the ζ-WGD were preferentially retained over duplicates derived from the gymnosperm-WGD in all the species surveyed. In addition, a *Ks* peak (~ 0.8) was observed recently in the *Cycas* genome, which is similar to the value for *Ginkgo* [25], suggesting a shared ancient WGD by the two lineages, as proposed by Roodt et al. (2017) [86]. This ancient WGD (named ω-) was further dated to the most recent common ancestors (MRCA) of all gymnosperms and supported by both transcriptome data and multispecies syntenic block alignment [25]. However, analysis with a probabilistic approach for WGD inference against 21 representative seed plants showed clear evidence of the ζ-WGD but not the ω-WGD, which made the placement of the *Cycas*+*Ginkgo* WGD highly controversial [23, 80] (Fig. 1C).

Given the considerable number of ancient WGDs predicted, at least based on the increased signals of gene duplication (restricted to WGD-derives) [14,17], the question was raised regarding how polyploidy contributes to the evolution of gymnosperms. A recent

comprehensive measurement of traits from living and fossil records suggested that two old-age pulsed rises of morphological innovation occurred in seed plants evolutionary history, including the incipient diversification of gymnosperms (ca. 400 Ma) and subsequent prosperity of angiosperms during the Late Cretaceous (ca. 100 Ma) [87]. The first increase represented by gymnosperms seems to be obedience to the most commonly shared ζ- WGD and can be extended to the hypothetical ω- WGD. Two direct correlations between the conifers' WGD and their diversification shifts [14] also likely suggest their potential roles in culminating of early gymnosperms (*Cupressophyta*-WGD and *Pinaceae*-WGD occurred ca. 200-342 Ma [85]). Then, considerable evolutionary stasis persisted regarding the morphological complexity of gymnosperms and was further exacerbated by the emergence of flowering plants [87]. One report linked to genetic map analysis displayed that many more ζ -duplicates (688 gene pairs) than conifer-specific tandem duplicates (87 pairs) were preserved in *Pinaceae* genomes. A highly conserved genome macrostructure was found between spruce and pine, which diverged at least 120 Ma ago [88]. The large excess of ancestral duplicates and remarkable level of synteny indicated the much slower pace of evolution in *Pinaceae*, which can be further interpreted as evidence of their relative stasis. Interestingly, karyotype comparison between *Pinaceae* and *Cupressaceae* suggested that substantial chromosomal shuffling likely occurred after their split [89]. Interspecies alignments within *Cupressaceae* and other families are called to determine if the shuffling is a common feature of low-frequency genome rearrangements, which would help our understanding of conifer cladogenesis resulting in speciation and diversity. More than that, a moderate case of coast redwood (*Sequoia sempervirens*) implied that there was a very slow diploidization process following WGD and the persistence of multi-somic inheritance in this hexaploidy species (2n = 66), which might contribute to why so few polyploid species occur in modern gymnosperms [90]. Normally, the long-term benefits of polyploidy require divergence among homologous chromosomes, which can only happen once loci are diploidized [78,90]. In turn, the reduced selection of efficient meiosis in *Sequoia* would preclude the emergence of any evolutionary advantages in polyploidy lineages. Hence, Scott et al. (2016) [90] further proposed that such an intriguing evolutionary strategy was additionally reinforced by asexual reproduction, self-compatibility and extreme longevity, which likely took place in other conifers, such as *Fizroya cupressoides* [91]. With regard to this, the fundamental dynamic shift in repeats is noteworthy, assuming that the genomic shift occurred early in gymnosperms, even probably before most of the modern lineages diverged. The ancestral genome size of gymnosperms has been estimated to be ~12.375-15.75 Gb [92]. If so, heterogeneous rates of genome size evolution should be expected considering the large range in 1C-DNA content (i.e., from 2.21 Gb in *G. ula* to 35.28 Gb in *P. ayacahuite*) exhibited across lineages [12] (**Fig. 1D and E**). The shift in genomic dynamics could directly lead to the unfavorable makeup of those large genomes as constrained chromosomal homogenization. Together with the slow pace of diploidization, these factors compulsively make polyploidy

more of a burden than a boon in gymnosperms. Therefore, the extraordinarily massive loss of duplicates should not be a surprise due to the highly structured chromosomes and severely limited recombination in these genomes [4]; hence, most signals of WGD in the doubled genome were expunged (e.g., to date, *Welwitschia* is the only gymnosperm species who is known to have a family-specific WGD occurring at ~86 Ma ago but shows an extremely low level of intrachromosomal syntenic relationships compared to angiosperms) [24]. The unusually low rate of WGD duplicate retention could further restrain the morphological and biological diversity of these lineages given that polyploidy often introduces sub- or neofunctionalization and increases variations in dosage-sensitive genes and pathways [93-95]. Beyond that, the concomitant problems imposed by an enlarged genome could affect diverse physiological processes of plants as a whole, such as longer cell cycle times [96,97] and higher nutrient costs [4], which eventually impact the competitiveness of species.

Intriguing intron morphology and evolution in gymnosperms

The presence of astonishingly long genes had been extensively reported in many gymnosperms along distinct lineages [8,12,20] (**Fig. 1C**). These long genes are often associated with large amounts of intronic sequences characterized by cumulative size distributions, including numerous atypical long ones (> 20 kb) [8,12,20,25]. Why these very long introns are preserved and how they influence gene evolution and function in gymnosperms remain largely unanswered [12].

It has long been acknowledged that genome size may be correlated with intron size across broad phylogenetic groups, yet such a pattern was translated not so well into some narrow taxonomic distance groups in angiosperms [98]. A pioneering description and comparison of gene structures between *P. glauca* and *P. taeda* with data from BAC clones and genome scaffolds indicated a related conserved signature in the position of long introns [26]. Moreover, a high frequency (32%) of TEs displayed in the captured sequences, even in introns < 1 kb, suggested an important role of such invasive elements in the long gene space [26]. Niu et al. (2022) [12] tabulated the characteristics of gene structures among 68 recently sequenced seed plants and found a positive correlation between the ratio of total intron/exon length and genome size, especially in gymnosperm lineages (**Fig. 1C**). Collectively, these robust evidence supports the claim that genic expansion was particularly coupled with the genome upsizing in the majority of gymnosperms, which is probably attributed to slow growth and the accumulation of repeats [12]. Additionally, Nystedt et al. (2013) [8] first provided insights into the presence of long introns by comparisons of orthologues of normal-sized (50-300 bp) and long (1-20 kb) introns in *P. abies, Pinus sylvestris*, and *G. montanum*. They suggested that early intron expansion might have occurred in the MRCA of all conifers, which would explain the identical trend in the increased length of orthologous introns. However, this point of view was considerably modified later by comparisons conducted among more species of early diverged

seed plants [21]. Similar growth patterns in both intron size and content were observed in orthologues between *Ginkgo* and *P. taeda* with the accumulation of LTR-RTs (especially Ty1-*copia* elements). By contrast, a high proportion of long interspersed nuclear elements (LINEs) were displayed in orthologous long introns between *G. montanum* and *Amborella trichopoda* (the 'basal' angiosperm [99]), and both of the manners involved the expansion of long introns, consistent with the scenario of all intron morphology in *G. montanum* and *A. trichopoda* [21]. This result might indicate different repeats dynamics within introns of *G. montanum* compared with other gymnosperms, and the level of Ty1-*Copia* activity in introns might be more ancient and could be traced back to the origin of gymnosperms. Likewise, LINEs could be partially involved in intron evolution in ancestral seed plants [21]. However, these hypotheses require more investigations using closely related or representative species like *Welwitschia*, *Ephedra*, and even *Cycads*, because the evolution of plant gene structure is determined by much more interacting forces than classically expected (i.e., selective recombination rate [100,101], species-specific TE activity [102,103]). Indeed, a large portion of unknown sequences has been found in *Cycas*' introns, which is quite different from the pattern of LTR or LINE dominance found in other gymnosperms [25].

Exploring the probable biological relevance of long introns could be insightful for understanding the famous scientific inquiry: "Why some genomes really big and others quite compact ? ". Unfortunately, it has been poorly interrogated in gymnosperms [26] except for a very recent description of the aspects of gene expression profiles, alternative splicing, and DNA methylation [12]. The atypical long introns seem to have minimal influences on transcript accuracy, probably facilitated by different levels of CpG and non-CpG methylation among exons and introns [12]. These results call for similar examinations in other giant gymnosperm genomes, such as *Ginkgo* or *Welwitschia*, considering their lower effective population size compared to conifers, since the loosening of natural selection often allows the fixation of potentially deleterious mutations in the genome [104]. In addition, long genes tend to have higher expression levels in *P. tabuliformis*, which is similar to the pattern in *P. glauca*, *Oryza sativa*, and *A. thaliana* [26,105]. The 'low-cost transcription hypothesis' is apparently not suitable here, alternatively, the length of the intron is probably less relevant to the level of expression as a whole since introns are involved in a variety of regulatory phenomena (i.e., post-transcriptional gene regulation [106], nucleosome formation and chromatin organization [107-109]).

Conclusion and perspectives

In this review, with a great appreciation of previous advances in the genome evolution of gymnosperms, we demonstrated that some essential characteristics, such as repeats dynamics, ancient WGDs inference, and the biological relevance of long introns, are far from understood. The state of 'genome paralysis' might be confined to *Pinaceae* rather than present in all conifers

or gymnosperms since a high frequency of TE removal does exist in *Cupressophytes*, *Gnetophytes*, and *Ginkgo*. The hypothetical ω-WGD is still highly contested and needs reconsideration with more studies. The sporadic and long-awaited releases of genome drafts inevitably limited the relevant conclusions to species-specific cases. Despite the low level of cladogenesis and the rarity of polyploids, the fundamental shift of genomic dynamics and a potential signature of the slow process of diploidization probably offer new insights into the complex evolution of gymnosperm genome architectures. Beyond that, the dominant model of recent allopolyploidy speciation in *Ephedra* [110], as well as the growing number on list of hybridization and polyploidization in *Juniperus* [111], collectively contrasts with the reputation of gymnosperms as ancient, relict species. These results could be appropriately addressed to the resurgence of gymnosperm diversification together with increases in habitat ranges [14]. With regard to all these aspects, we envisaged that gymnosperms could be a candidate model to investigate the changes in genome dynamics and their influences on subsequent species diversification (**Fig. 1E**). However, in-depth studies on the wealth of information contained within the genomes cannot be conducted without multiple high-quality assemblies. The investigation of interspecific variation and diverse properties in gymnosperms would be much profound if the data sampled were of relatively consistent quality, as in many excellent works conducted in animals or crops [**112,113**].

Considering the intricate evolutionary history of gymnosperms, we propose that in the future attention should be given to at least four aspects. First, more integrative estimations of TE elimination are needed, and high-resolution subclassification of TEs would help to distinguish family-specific expansion patterns. Intensive studies on the large amount of low copy number repetitive relics would also enable us to better illustrate the formation of the highly-structured and less dynamic chromosomes of gymnosperms [4,8,72]. Rapid accumulation of epigenetic data is imperative either at the single-base resolution of DNA methylation or for comparative methylomes among different tissues since variable repeats dynamic and sophisticated epigenetic machinery are involved in gymnosperms. Second, ancestral paleopolylpoidy inferences should be investigated by large-scale multi-alignments among more complete gymnosperm assemblies with fully considered phylogenies. Structural evidence of intra- and interspecies collinearity may be essential to clarify the number and timing of these ancient duplications [79]. Moreover, the comprehensive evaluation of the loss and retention of duplicate genes could help to elucidate the potential heterogeneity in the genome evolution of gymnosperms. Third, it may be worthwhile to include intron length and expression characteristics in future whole genome studies of gymnosperms. More investigations on alternative splicing complexity should be carried out and addressed together with DNA methylation footprints. Despite the lack of appropriate genetic transformation tools for typical long-lived perennial species, it might be insightful to conduct analogous molecular experiments in model plant systems concerning the potential biological functions of ultra-long genes. Finally,

more chromosome-level genomes of gymnosperms are always needed, but we suggest that additional efforts should be made towards medium-sized (5-15 G) species as well as the refinement of short-read drafts released for conifers, especially *Pinaceae*.

Competing interests

The authors declare no competing interests.

Funding

This work was supported by the Scientific Research Program of Sino-Africa Joint Research Center (grant no. SAJC202105), the National Natural Science Foundation of China grants (grant no. 31870206)

Author contributions

T.W and Q.F.W designed the outline of the manuscript. T.W., Y.B.G wrote the manuscript. T.W., C.D and Q.F.W polished the article. Z.M.L. and Y.D.Z. worked on the revisitation of genomic data. T.W. and Y.B.G are joint first authors.

Acknowledgments

We wish to thank Dr. Neng Wei from Wuhan Botanical Garden for the collection of species images. And acknowledge Prof. Shouzhou Zhang from Fairy Lake Botanical Garden for the assistance of data access to the *Cycas* genome assembly. No conflict of interest declared.

References

1. Initiative TAG. Analysis of the genome sequence of the flowering plant *Arabidopsis thaliana*. *Nature* 2000; **408**:796-815.
2. Marks RA, Hotaling S, Frandsen PB, VanBuren R. Representation and participation across 20 years of plant genome sequencing. *Nat Plants* 2021; **7**:1571-1578.
3. Murray BG. Nuclear DNA amounts in gymnosperms. *Ann Bot.* 1998; **82**: 3-15.
4. Leitch AR, Leitch IJ. Ecological and genetic factors linked to contrasting genome dynamics in seed plants. *New Phytol* 2012; **194**: 629-646.
5. Sederoff R. Genomics: A spruce sequence. *Nature* 2013; **497**: 569-570.
6. Neale DB, Zimin AV, Zaman S, Scott AD, Shrestha B, Workman RE, et al. Assembled and annotated 26.5 Gbp coast redwood genome: a resource for estimating evolutionary adaptive potential and investigating hexaploid origin. G3 (Bethesda)**12**: jkab380.
7. Jin W-T, et al. Phylogenomic and ecological analyses reveal the spatiotemporal evolution of global pines. *Proc Natl Acad Sci USA.* 2021; **118** (20): e2022302118.
8. Nystedt B, et al. The Norway spruce genome sequence and conifer genome evolution. *Nature* 2013; **497**: 579-584.
9. Birol I, et al. Assembling the 20 Gb white spruce (*Picea glauca*) genome from whole-genome shotgun sequencing data. *Bioinformatics* 2013; **29**: 1492-1497.

10. Neale DB, et al. Decoding the massive genome of loblolly pine using haploid DNA and novel assembly strategies. *Genome Biol* 2014; **15** (3): R59.
11. Stevens KA, et al. Sequence of the Sugar Pine megagenome. *Genetics* 2016; **204**: 1613-1626.
12. Niu S-H, et al. The Chinese pine genome and methylome unveil key features of conifer evolution. *Cell* 2022; **185**: 1-14.
13. Warren RL, et al. Improved white spruce (*Picea glauca*) genome assemblies and annotation of large gene families of conifer terpenoid and phenolic defense metabolism. *Plant J* 2015; **83**: 189-212.
14. Stull GW, et al. Gene duplications and phylogenomic conflict underlie major pulses of phenotypic evolution in gymnosperms. *Nat. Plants* 2021; **7**: 1015-1025.
15. Wickett NJ, et al. Phylotranscriptomic analysis of the origin and early diversification of land plants. *Proc Natl Acad Sci USA* 2014; **111**: 4859-4868.
16. Ran J-H, Shen T-T, Wang M-M, Wang X-Q. Phylogenomics resolves the deep phylogeny of seed plants and indicates partial convergent or homoplastic evolution between Gnetales and angiosperms. *Proc R Soc B* 2018; **285** (1881): 20181012.
17. Leebens-Mack JH, et al. One thousand plant transcriptomes and the phylogenomics of green plants. *Nature* 2019; **574**: 679–685.
18. Li H-T, et al. Origin of angiosperms and the puzzle of the Jurassic gap. *Nat Plants* 2019; **5**: 461-470.
19. Song C, et al. *Taxus yunnanensis* genome offers insights into gymnosperm phylogeny and taxol production. *Commun Biol* 2021; **4**: 1203.
20. Guan R, et al. Draft genome of the living fossil *Ginkgo biloba*. *Gigascience* 2016; **5**: 49.
21. Wan T. et al. A genome for gnetophytes and early evolution of seed plants. *Nat Plants* 2018; **4**: 82-89.
22. Zhao Y-P, et al. Resequencing 545 ginkgo genomes across the world reveals the evolutionary history of the living fossil. *Nat Commun* 2019; **10**: 4201.
23. Liu H-L, et al. The nearly complete genome of *Ginkgo biloba* illuminates gymnosperm evolution. *Nat Plants* 2021; **7**: 748-756.
24. Wan T, et al. The *Welwitschia* genome reveals a unique biology underpinning extreme longevity in deserts. *Nat Commun* 2021; **12**: 4247.
25. Liu Y, et al. The *Cycas* genome and the early evolution of seed plants. *Nat Plants* 2022; **8**: 389-401.
26. Sena JS, et al. Evolution of gene structure in the conifer *Picea glauca*: a comparative analysis of the impact of intron size. *BMC Plant Biology* 2014; **14**: 95.
27. Van Dijk, E L, Auger H, Jaszczyszyn Y, Thermes C. Ten years of next-generation sequencing technology. *Trends Genet* 2014; **30**: 418-426.
28. Myers EW. Toward simplifying and accurately formulating fragment assembly. *J Comput Biol.* 1995; **2**: 275-290.
29. Li R-Q, et al. The sequence and *de novo* assembly of the giant panda genome. *Nature* 2010; **463**: 311-317.
30. Birol I, Raymond A, Jackman SD, Pleasance S, Coope R, et al. Assembling the 20 Gb white spruce (*Picea glauca*) genome from whole-genome shotgun sequencing data. Bioinformatics 2013; **29**:1492-1497.
31. Zimin A, et al. Sequencing and assembly of the 22-Gb loblolly pine genome. *Genetics* 2014; **196**: 875-890.
32. Kuzmin DA, Feranchuk SI, Sharov VV, Cybin AN, Makolov SV, et al. Stepwise large genome

assembly approach: a case of Siberian larch (*Larix sibirica* Ledeb.). BMC Bioinformatics 2019; **20**:37.

33. Neale DB, McGuire PE, Wheeler NC, Stevens KA, Crepeau MW, et al. The Douglas-fir genome sequence reveals specialization of the photosynthetic apparatus in Pinaceae. G3 (Bethesda) 2017; **7**: 3157–3167.
34. Mosca E, Cruz F, Gómez Garrido J, Bianco L, Rellstab C, et al. A reference genome sequence for the European silver fir (*Abies alba* Mill.): a community-generated genomic resource. G3 (Bethesda) 2019; **9:**2039–2049.
35. Scott AD, Zimin AV, Puiu D, Workman R, Britton M, et al. A reference genome sequence for giant sequoia. G3 (Bethesda) 2020; **10**:3907–3919.
36. Cheng J, et al. Chromosome-level genome of Himalayan yew provides insights into the origin and evolution of the paclitaxel biosynthetic pathway. *Molecular Plant* 2021; **14**: 1199-1209.
37. Xiong X-Y, et al. The *Taxus* genome provides insights into paclitaxel biosynthesis. *Nat Plants* 2021; **7**:1026-1036.
38. Seppey M, Manni M, Zdobnov EM. BUSCO: assessing genome assembly and annotation completeness. *Methods Mol Biol* 2019; **1962**:227-245.
39. Meyer A. et al. Giant lungfish genome elucidates the conquest of land by vertebrates. *Nature* 2021; **590**:284-289.
40. Ma J-X, Devos KM, Bennetzen JL. Analyses of LTR-retrotransposon structures reveal recent and rapid genomic DNA loss in rice. *Genome Res* 2004; **14**:860-869.
41. Lim KY, et al. Sequence of events leading to near-complete genome turnover in allopolyploid *Nicotiana* within five million years. *New Phytol* 2007; **175**:756-763.
42. Kejnovsky E, Leitch IJ, Leitch AR. Contrasting evolutionary dynamics between angiosperm and mammalian genomes. *Trends Ecol Evol* 2009; **24**:572-582.
43. Kumar A, Bennetzen JL. Plant retrotransposons. *Annu Rev Genet* 1999; **33**: 479-532.
44. Moffat AS. Transposons help sculpt a dynamic genome. *Science* 2000; **289**: 1455-1457.
45. Feschotte C, Jiang N, Wessler SR. Plant transposable elements: where genetics meets genomics. *Nat Rev Genet* 2002; **3**:329-341.
46. Vitte C, Panaud O, Quesneville H. LTR retrotransposons in rice (*Oryza sativa*, L.): recent burst amplifications followed by rapid DNA loss. *BMC Genet* 2007; **8**:218.
47. Devos KM, Brown JKM, Bennetzen JL. Genome size reduction through illegitimate recombination counteracts genome expansion in *Arabidopsis*. *Genome Res* 2002; **12**:1075-1079.
48. Vicient CM. et al. Retrotransposon BARE-1 and its role in genome evolution in the genus Hordeum. *Plant Cell* 1999; **11**:1769-1784.
49. Cossu RM, et al. LTR retrotransposons show low levels of unequal recombination and high rates of intraelement gene conversion in large plant genomes. *Genome Biol Evol* 2017; **9**:3449-3462.
50. Kelly LJ. et al. Analysis of the giant genomes of *Fritillaria* (Liliaceae) indicates that a lack of DNA removal characterizes extreme expansions in genome size. *New Phytol* 2015; **208**:596-607.
51. Vu GTH, Cao H-X, Reiss B, Schubert I. Deletion-bias in DNA double-strand break repair differentially contributes to plant genome shrinkage. *New Phytol* 2017; **214**:1712-1721.
52. Kim JH, Ryu TH, Lee SS, Lee S, Chung B-Y. Ionizing radiation manifesting DNA damage response in plants: an overview of DNA damage signaling and repair mechanisms in plants. *Plant Sci* 2019; **278**:44-53.
53. Block-Schmidt AS, Dukowic-Schulze S, Wanieck K, Reidt W, Puchta H. BRCC36A is epistatic to

BRCA1 in DNA crosslink repair and homologous recombination in *Arabidopsis thaliana*. *Nucleic Acids Res* 2011; **39**:146-154.

54. Fedoroff NV. Transposable elements, epigenetics, and genome evolution. *Science* 2012; **338**:758-767.
55. Barghini E. et al. LTR retrotransposon dynamics in the evolution of the olive (*Olea europaea*) genome. *DNA Res* 2015; **22**:91-100.
56. Edgar RC. MUSCLE: multiple sequence alignment with high accuracy and high throughput. *Nucleic Acids Res* 2004; **32**:1792-1797.
57. De La Torre AR, Li Z, Van de Peer Y, Ingvarsson PK. Contrasting rates of molecular evolution and patterns of selection among gymnosperms and flowering plants. *Mol Biol Evol* 2017; **34**:1363-1377.
58. Morse AM. et al. Evolution of genome size and complexity in *Pinus*. *PLoS One* 2009; **4**:e4332.
59. Zhou S-S, et al. A comprehensive annotation dataset of intact LTR retrotransposons of 300 plant genomes. *Sci Data* 2021; **8**:174.
60. Zhou W-D, Liang G-N, Molloy PL, Jones PA. DNA methylation enables transposable element-driven genome expansion. *Proc Natl Acad Sci USA* 2020; **117**:19359-19366.
61. Finnegan EJ, Peacock WJ, Dennis ES. Reduced DNA methylation in *Arabidopsis thaliana* results in abnormal plant development. *Proc Natl Acad Sci USA* 1996; **93**:8449-8454.
62. Jeddeloh JA, Stokes TL, Richards EJ. Maintenance of genomic methylation requires a SW12/SNF2-like protein. *Nat Genet* 1999; **22**:94-97.
63. Zemach A. et al. The *Arabidopsis* nucleosome remodeler DDM1 allows DNA methyltransferases to access H1-containing heterochromatin. *Cell* 2013; **153**:193-205.
64. Ito H, Kakutani T. Control of transposable elements in *Arabidopsis thaliana*. *Chromosome Res* 2014; **22**:217-223.
65. Matzke MA, Mosher RA. RNA-directed DNA methylation: an epigenetic pathway of increasing complexity. *Nat Rev Genet* 2014; **15**:394-408.
66. Wassenegger M, Heimes S, Riedel L, Sanger HL. RNA-directed de novo methylation of genomic sequences in plants. *Cell* 1994; **76**:567-576.
67. Ausin I, et al. DNA methylome of the 20-gigabase Norway spruce genome. *Proc Natl Acad Sci USA* 2016; **113**:e8106-e8113.
68. Takuno S, Ran J-H, Gaut BS. Evolutionary patterns of genic DNA methylation vary across land plants. *Nat Plants* 2016; **2**(2):15222.
69. Zhang H-M, Zhu J-K. RNA-directed DNA methylation. *Curr Opin Plant Biol* 2011; **14**:142-147.
70. Fuchs J, Jovtchev G, Schubert I. The chromosomal distribution of histone methylation marks in gymnosperms differs from that of angiosperms. *Chromosome Res* 2008; **16**:891-898.
71. Nóvak P. et al. Repeat-sequence turnover shifts fundamentally in species with large genomes. *Nat Plants* 2020; **6**:1325-1329.
72. Islam-Faridi MN, Nelson CD, Kubisiak TL. Reference karyotype and cytomolecular map for loblolly pine (*Pinus taeda* L.). *Genome* 2007; **50**:241-251.
73. Bennetzen JL, Kellogg EA. Do plants have a one-way ticket to genomic obesity ? *Plant Cell* 1997; **9**:1509-1514.
74. Elsik CG, Williams CG. Retroelements contribute to the excess low-copy-number DNA in pine. *Mol Gen Genet* 2000; **264**:47-55.
75. Jiao Y-N, et al. Ancestral polyploidy in seed plants and angiosperms. *Nature* 2011; **473**:97-100.
76. Soltis PS, Soltis DE. Ancient WGD events as drivers of key innovations in angiosperms. *Curr Opin*

Plant Biol 2016; **30**:159-165.
77. Wu S-D, Han B-C, Jiao Y-N. Genetic contribution of paleopolyploidy to adaptive evolution in angiosperms. *Molecular Plant* 2020; **13**:59-71.
78. Mandakova T, Lysak MA. Post-polyploid diploidization and diversification through dysploid changes. *Curr Opin Plant Biol* 2018; **42**:55-65.
79. Ruprecht C, et al. Revisiting ancestral polyploidy in plants. *Sci. Adv* 2017; **3**:1603195.
80. Zwaenepoel A, Van de Peer Y. Inference of ancient whole-genome duplications and the evolution of gene duplication and loss rates. *Mol Biol Evol* 2019; **36**:1384-1404.
81. Lynch M, Conery JS. The evolutionary fate and consequences of duplicate genes. *Science* 2000; **290**:1151-1155.
82. Blanc G, Wolfe KH. Widespread paleopolyploidy in model plant species inferred from age distributions of duplicate genes. *Plant Cell* 2004; **16**:1667-1678.
83. Rabier CE, Ta T, Ane C. Detecting and locating whole genome duplications on a phylogeny: a probabilistic approach. *Mol Biol Evol* 2014; **31**:750-762.
84. Yang Y, et al. Improved transcriptome sampling pinpoints 26 ancient and more recent polyploidy events in Caryophyllales, including two allopolyploidy events. *New Phytol* 2018; **217**:855-870.
85. Li Z, et al. Early genome duplications in conifers and other seed plants. *Sci Adv* 2015; **1**:e1501084.
86. Roodt D, et al. Evidence for an ancient whole genome duplication in the cycad lineage. *PLoS One* 2017; **12**:e0184454.
87. Leslie AB, Simpson C, Mander L. Reproductive innovations and pulsed rise in plant complexity. *Science* 2021; **373**:1368-1372.
88. Pavy N, et al. A spruce gene map infers ancient plant genome reshuffling and subsequent slow evolution in the gymnosperm lineage leading to extant conifers. *BMC Biol* 2012; **10**:84.
89. De Miguel M, et al. Evidence of intense chromosomal shuffling during conifer evolution. *Genome Biol Evol* 2015; **7**:2799-2809.
90. Scott AD, Stenz NWM, Ingvarsson PK, Baum DA. Whole genome duplication in coast redwood (*Sequoia sempervirens*) and its implications for explaining the rarity of polyploidy in conifers. *New Phytol* 2016; **211**:186-193.
91. Silla F, Fraver S, Lara A, Allnutt TR, Newton A. Regeneration and stand dynamics of *Fitzroya cupressoides* (Cupressaceae) forests of southern Chile's Central Depression. *For Ecol Manage* 2002; **165**:213-224.
92. Burleigh JG, Barbazuk WB, Davis JM, Morse AM, Soltis PS. Exploring diversification and genome size evolution in extant gymnosperms through phylogenetic synthesis. *J Bot* 2012; **2012**: 292857.
93. Freeling M. Bias in plant gene content following different sorts of duplication: tandem, whole-genome, segmental, or by transposition. *Annu Rev Plant Biol* 2009; **60**:433-453.
94. Bekaert M, Edger PP, Pires JC, Conant GC. Two-phase resolution of polyploidy in the *Arabidopsis* metabolic network gives rise to relative and absolute dosage constraints. *Plant Cell* 2011; **23**:1719-1728.
95. Conant GC, Birchler JA, Pires JC. Dosage, duplication, and diploidization: clarifying the interplay of multiple models for duplicate gene evolution over time. *Curr Opin Plant Biol* 2014; **19**:91-98.
96. Francis D, Davies MS, Barlow PW. A strong nucleotypic effect on the cell cycle regardless of ploidy level. *Ann Bot* 2008; **101**:747-757.
97. Doyle JJ, Coate JE. Polyploidy, the nucleotype, and novelty: the impact of genome doubling on the biology of the cell. *Int J Plant Sci* 2019; **180**:1-52.

98. Wendel JF, et al. Intron size and genome size in plants. *Mol Biol Evol* 2002; **19**:2346-2352.
99. Amborella Genome Project. The *Amborella* genome and the evolution of flowering plants. *Science* 2013; **342**:1241089.
100. Carvalho AB, Clark AG. Intron size and natural selection. *Nature* 1999; **401**:344-344.
101. Comeron JM, Kreitman M. The correlation between intron length and recombination in *Drosophila*: Dynamic equilibrium between mutational and selective forces. *Genetics* 2000; **156**:1175-1190.
102. Vinogradov AE. Intron-genome size relationship on a large evolutionary scale. *J Mol Evol* 1999; **49**:376-384.
103. McLysaght A, Enright AJ, Skrabanek L, Wolfe KH. Estimation of synteny conservation and genome compaction between pufferfish (Fugu) and human. *Yeast* 2000; **17**:22-36.
104. Lynch M. Intron evolution as a population-genetic process. *Proc Natl Acad Sci USA* 2002; **99**:6118-6123.
105. Ren X-Y, Vorst O, Fiers M, Stiekema WJ, Nap JP. In plants, highly expressed genes are the least compact. *Trends Genet* 2006; **22**:528-532.
106. Shabalina SA, Spiridonov NA. The mammalian transcriptome and the function of non-coding DNA sequences. *Genome Biol* 2004; **5**:105.
107. Zuckerkandl E. Junk DNA and sectorial gene repression. *Gene* 1997; **205**:323-343.
108. Mattick JS, Gagen MJ. The evolution of controlled multitasked gene networks: the role of introns and other noncoding RNAs in the development of complex organisms. *Mol Biol Evol* 2001; **18**:1611-1630.
109. Vinogradov AE. Noncoding DNA, isochores and gene expression: nucleosome formation potential. *Nucleic Acids Res* 2005; **33**:559-563.
110. Wu H, et al. A high frequency of allopolyploid speciation in the gymnospermous genus *Ephedra* and its possible association with some biological and ecological features. *Mol Ecol* 2016; **25**:1192-1210.
111. Farhat P, et al. Polyploidy in the conifer genus *Juniperus*: an unexpectedly high rate. *Front Plant Sci* 2019; **10**:676.
112. Zhang GJ, et al. Comparative genomics reveals insights into avian genome evolution and adaptation. *Science* 2014; **346**:1311-1320.
113. Varshney RK, et al. A chickpea genetic variation map based on the sequencing of 3,366 genomes. *Nature* 2011; **599**:622-627.

Table 1. The list of currently available whole-genome assembly of gymnosperms

Species	Size of Assembly (bp)	Family	Sequencing Platform	Online Year
Picea abies	12.3 G	Pinaceae	Sanger whole-genome shotgun	2013
Picea glauca (genotype PG29)	23.6 G	Pinaceae	Illumina HiSeq 2000, Miseq	2013
Pinus taeda	22 G	Pinaceae	Sanger+ Illumina HiSeq 2000	2014
Pinus taeda (genotype 20-1010)	22.6 G	Pinaceae	Illumina GA II, HiSeq 2000, Miseq	2014
Picea glauca (genotype WS77111)	22.4 G	Pinaceae	Illumina HiSeq2500, MiSeq	2015
Pinus lambertiana	27.6 G	Pinaceae	Illumina GA II, HiSeq 2000/2500, Miseq	2016
Ginkgo biloba	10.6 G	Ginkgoaceae	Illumina Hiseq 2000/4000	2016
Pseudotsuga menziesii	15.7 G	Pinaceae	Illumina HiSeq	2017
Gnetum montanum	4.0 G	Gnetaceae	Illumina HiSeq 2000/2500	2018
Abies alba	18.2 G	Pinaceae	Illumina HiSeq	2019
Larix sibirica	12.3 G	Pinaceae	Illumina HiSeq	2019
Sequoiadendron giganteum	8.1 G	Cupressaceae	Illumina HiSeq + Oxford Nanopore	2020
Ginkgo biloba	9.8 G	Ginkgoaceae	Illumina HiSeq + PacBio RSII	2021
Welwitschia mirabilis	6.8 G	Welwitschiaceae	Illumina HiSeq + Oxford Nanopore	2021
Taxus chinensis	10.2 G	Taxaceae	Illumina HiSeq + PacBio RSII	2021
Taxus wallichiana	10.9 G	Taxaceae	Illumina HiSeq + Oxford Nanopore	2021
Taxus yunnanensis	10.7 G	Taxaceae	Illumina HiSeq + Oxford Nanopore	2021
Pinus tabuliformis	25.4 G	Pinaceae	Illumina HiSeq + PacBio RSII	2022
Sequoia sempervirens	26.5 G	Cupressaceae	Illumina HiSeq + Oxford Nanopore	2022
Cycas panzhihuaensis	10.5 G	Cycadaceae	Illumina HiSeq, Miseq+ Oxford Nanopore	2022

Figure 1: The contemporary overview of deciphered gymnosperms genome and genomic features underpinning their complicated evolutionary history. (A) The geographical distribution of extant gymnosperms was depicted based on Global Biodiversity Information Facility (GBIF). The images listed the representative gymnosperm species that have been sequenced. **(B)** The growing wealth of high-quality assemblies on the advent of long-read sequencing technology and great efforts to make taxonomic samples fully covered. The abbreviation of taxon listed in order from top to bottom as: Pab = *Picea abies*, Pgl = *Picea glauca*, Pta = *Pinus taeda*, Pla = *Pinus lambertiana*, Gbi = *Ginkgo biloba*, Pme = *Pseudotsuga menziesii*, Gmo = *Gnetum montanum*, Aal = *Abies alba*, Sgi = *Sequoiadendron giganteum*, Wmi= *Welwitschia mirabilis*, Tyu= *Taxus yunnanensis*, Sse = *Sequoia sempervirens*, Ptab = *Pinus tabuliformis*, Cpa = *Cycas panzhihuaensis*. **(C)** The prediction and placement of ancient WGDs in seed plants and the highly contested inference of paleopolyploidy in MRCA of all extant gymnosperms. The dashed line indicated the conflicts in phylogenetic position of *Gnetophytes.* The dash arrows referred to the controversy on the shared polyploidy event in gymnosperms. The Cupressaceae-WGD was highlighted with '*' since only *Taxus* and *Sequoiadendron* were included (excluding Araucaceae) as representatives of whole *cupressophytes* in the sketched tree (left). The available records of the ratio of solo-/intact LTR and relevance of intron length were accordingly mapped to the species (right). The data for estimation of solo-/intact LTR is from *Nystedt, et al.*, 2013, *Cossue, et al.*, 2017, *Wan, et al.*, 2018, *Cheng et al.*, 2021, *Wan, et al*., 2021, *Niu, et al*., 2022. The data on gene structure is from *Niu, et al.*, 2022. **(D)** The distribution of genome size along gymnosperm lineages with "medium-size" and "ultra-large size" distinguished. The counts on 1 C-DNA content were collected from *Niu et al., 2021,* and data sources of *Kew*. **(E)** The genomic signatures of gymnosperms and probable genome evolutionary pattern were summarized with recent advances both on recombination and repeats dynamic. Abbreviations: TEs = transposable elements; UR = unequal recombination; GCE = gene conversion event.

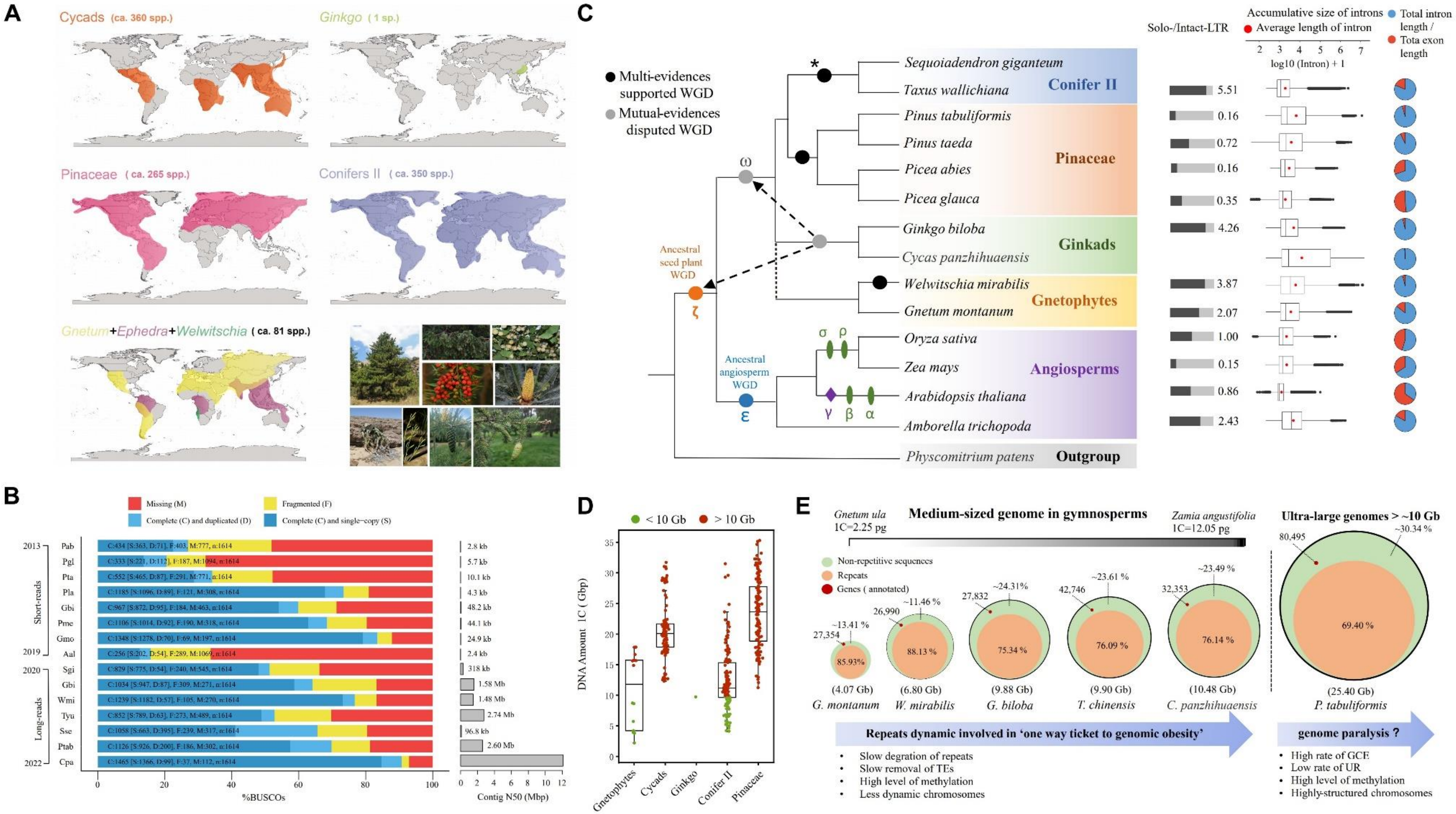
A
Cycads (ca. 360 spp.)
Ginkgo (1 sp.)
Pinaceae (ca. 265 spp.)
Conifers II (ca. 350 spp.)
Gnetum+Ephedra+Welwitschia (ca. 81 spp.)
B
Missing (M)
Fragmented (F)
Complete (C) and duplicated (D)
Complete (C) and single-copy (S)
2013
2019
2020
2022
Short-reads
Long-reads
Pab
Pgl
Pta
Pla
Gbi
Pme
Gmo
Aal
Sgi
Gbi
Wmi
Tyu
Sse
Ptab
Cpa
2.8 kb
5.7 kb
10.1 kb
4.3 kb
48.2 kb
44.1 kb
24.9 kb
2.4 kb
318 kb
1.58 Mb
1.48 Mb
2.74 Mb
96.8 kb
2.60 Mb
%BUSCOs
Contig N50 (Mbp)
C
Multi-evidences supported WGD
Mutual-evidences disputed WGD
Ancestral seed plant WGD
Ancestral angiosperm WGD
ω
ζ
ε
σ ρ
γ β α
Solo-/Intact-LTR
Accumulative size of introns
Average length of intron
log10 (Intron) + 1
Total intron length / Tota exon length
Sequoiadendron giganteum
Taxus wallichiana
Conifer II
Pinus tabuliformis
Pinus taeda
Pinaceae
Picea abies
Picea glauca
Ginkgo biloba
Cycas panzhihuaensis
Ginkads
Welwitschia mirabilis
Gnetum montanum
Gnetophytes
Oryza sativa
Zea mays
Angiosperms
Arabidopsis thaliana
Amborella trichopoda
Physcomitrium patens
Outgroup
5.51
0.16
0.72
0.16
0.35
4.26
3.87
2.07
1.00
0.15
0.86
2.43
D
< 10 Gb
> 10 Gb
DNA Amount 1C (Gbp)
Gnetophytes
Cycads
Ginkgo
Conifer II
Pinaceae
E
Gnetum ula 1C=2.25 pg
Medium-sized genome in gymnosperms
Zamia angustifolia 1C=12.05 pg
Ultra-large genomes > ~10 Gb
Non-repetitive sequences
Repeats
Genes (annotated)
27,354
~13.41 %
85.93%
26,990
~11.46 %
88.13 %
27,832
~24.31%
75.34 %
42,746
~23.61 %
76.09 %
32,353
~23.49 %
76.14 %
80,495
~30.34 %
69.40 %
(4.07 Gb) G. montanum
(6.80 Gb) W. mirabilis
(9.88 Gb) G. biloba
(9.90 Gb) T. chinensis
(10.48 Gb) C. panzhihuaensis
(25.40 Gb) P. tabuliformis
Repeats dynamic involved in 'one way ticket to genomic obesity'
Slow degration of repeats
Slow removal of TEs
High level of methylation
Less dynamic chromosomes
genome paralysis ?
High rate of GCE
Low rate of UR
High level of methylation
Highly-structured chromosomes

Click here to access/download;Figure;Figure 1.pdf

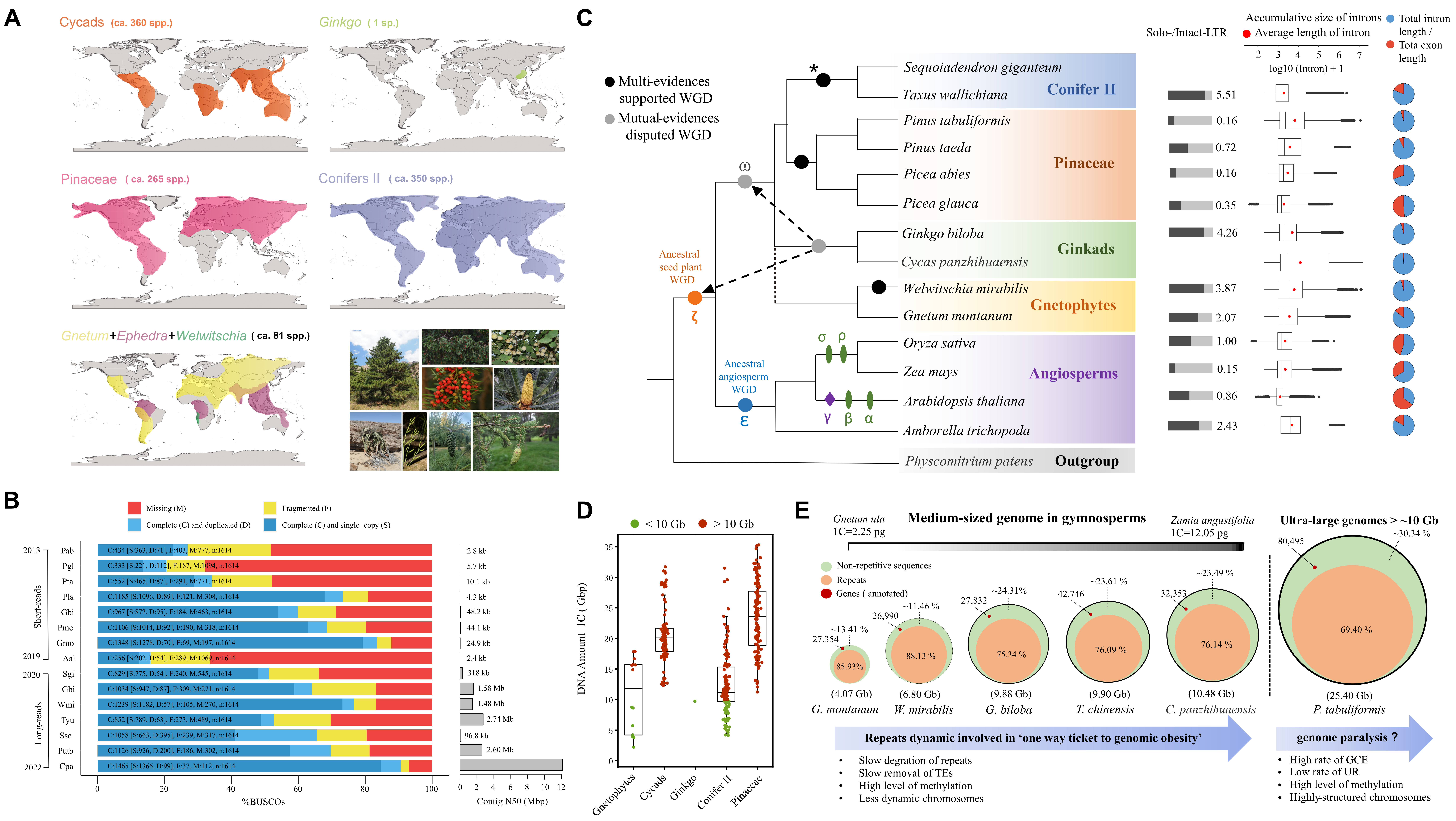

Supplement: giac078_GIGA-D-22-00123_Original_Submission [file giac078_giga-d-22-00123_original_submission.pdf]
