# Supplementary material for: Evolution of complex genome architecture in gymnosperms
Source: Gigascience. 2022 Aug 10;11:giac078. doi: 10.1093/gigascience/giac078 (PMC9364684; doi:10.1093/gigascience/giac078)
Supplement: giac078_Response_to_Reviewer_Comments_Original_Submission [file giac078_response_to_reviewer_comments_original_submission.pdf]

Author's Response To Reviewer Comments

Close

In this revised manuscript, we revisit all the concerns raised by referees and made the appropriate corrections. The paper has been polished by a native speaker to make it more readable. Along with our revised manuscript, we also wrote a point-by-point response letter to all of the questions and suggestions from the referees, with detailed notes of the changes that have been highlighted in the revised manuscript.

Close
