# Supplementary material for: Evolution of complex genome architecture in gymnosperms
Source: Gigascience. 2022 Aug 10;11:giac078. doi: 10.1093/gigascience/giac078 (PMC9364684; doi:10.1093/gigascience/giac078)
Supplement: giac078_Reviewer_1_Report_Original_Submission — Steven L. Salzberg, Ph.D. -- 1/15/2022 Reviewed [file giac078_reviewer_1_report_original_submission.pdf]

Reviewer Report

Title: Evolution of complex genome architecture in gymnosperms

Version: Original Submission **Date:** 6/1/2022

Reviewer name: Steven L. Salzberg, Ph.D.

Reviewer Comments to Author:

Review of Complexity of giant genome evolution in gymnosperms, Tao Wan et al.
This report presents an interesting review of the giant genomes sequenced from the phylogenetic grouping of trees known as gymnosperms, many of which are conifers. These genomes stand out for their unusually large size and very high repetitive content, which derives from an ancient genome expansion shared by all members of this group.
Major issues:
1. The authors describe the Norway spruce genome published in 2013 as "a milestone report" that "presented a 20-Gb genome" (p. 1, bottom). This is simply not correct. What happened was that the first publicly available conifer assembly was that of loblolly pine (Pinus taeda), for which a preliminary assembly containing 23 Gb was released in early 2013 (and an earlier assembly was released in 2012), well before the Norway spruce genome was published. The Norway spruce group rushed out a paper based on an assembly that contained only 12 Gbp, which was far from complete. They called it a 20-gigabase genome" in their abstract, but in fact Table 1 of that paper shows that the assembly only had 12 Gbp in scaffolds, which includes gaps. It's also an extremely fragmented assembly, with over 11 million scaffolds and an N50 size of just 3 Kb. This quite poor assembly was never improved; I checked the record at https://www.ncbi.nlm.nih.gov/assembly/GCA_900067695.1, and it hasn't even been updated since 2013. Thus it was a poor-quality, incomplete draft with only 60% of the genome present, not even a draft, but they rushed it to press so that they could claim credit for the first conifer genome, because they were well aware of the already-released data for loblolly pine, which was far more complete even in early 2013.
Thus in order to be accurate, the authors need to explain that the first near-complete draft genome to be made available was that of loblolly pine, for which a draft genome was released in 2012 and a paper published in 2014. The 2014 paper was the first conifer genome that one could call substantially complete, since it contained more than 90% of the genome (22 Gb in scaffolds). Not only was it far more complete, but it was also far more contiguous, with an N50 scaffold size of 62.7 Kb, over 20 times larger than the Norway spruce assembly. Otherwise this review paper will implicitly be stating that 60% of a genome is enough to claim precedence, which I'm sure the authors don't intend to do.
2. Table 1 is quite useful, but in a review like this it can be much more useful with a few additions. First, the authors would add the common names of all the trees, to appeal to a wider audience. Second, they should add links to (a) the publications for each genome (for those that are published), and (b) to the assemblies themselves, which all should be publicly available. Also note, as stated above, that Pinus taeda (loblolly pine) was available online in 2013, so the table should reflect that (right now it says 2014).

3. Bottom of p. 2: the authors cite "BUSCO values of 56.92%" as evidence for the low quality of gymnosperm genomes. Regardless of what you think of the assembly quality, BUSCO analysis is a very poor measure. First, the BUSCO gene set was designed for other species, and even the plant-specific set probably does not represent conifers very well. Second, the BUSCO software uses a variety of steps that can easily miss genes that are present in an assembly, especially an assembly that is relatively fragmented, and further it can mistakenly report genes are present in multiple copies when they are not. If the authors want to cite BUSCO statistics, they need to explain what it is and why they think it's a good metric for conifers. But they need to include some caveats.

4. The authors are wrong in their speculative statement that: "The Loblolly Pine Genome Project' was probably initiated in 1995, but data from this project was not publicly available until 2014 with rounds of sequencing data supply [10]." This is simply false. This reviewer was part of the loblolly project, which was launched with a USDA grant awarded in 2011. No one dreamed of attempting the whole genome until NGS technology was invented and read lengths got long enough to attempt it. The loblolly project was subsequently the first ever to release a whole-genome assembly, which as stated above and as reported at the time in multiple venues, was first made available in 2012 and then in an improved version in 2013, both prior to publication. This statement in the manuscript makes it seem that the loblolly project, which set a high standard for open sharing of data, was keeping data private, which was never the case.

Indeed, on this topic the authors should read and cite the paper by the loblolly genome project leaders about open access to tree genomes, published in 2013, which they can find here: https://genomebiology.biomedcentral.com/articles/10.1186/gb-2013-14-6-120. Table 1 in that paper contains a list of conifer genome projects and shows their status at that time, with references to publications where available. Notice that the Norway spruce genome, at the time this paper appeared, was "restricted" meaning that the website did not permit others to download it. That genome was not released until publication, unlike Pinus taeda or the other conifers sequenced by the Loblolly Pine Genome Project.

Minor issues:

The sentence (p. 3) doesn't make sense and needs to be rewritten or else removed: "Similar tough jobs underwent in the other conifer species including Douglas-fir (Pseudotsuga menziesii) [33] and silver fir (Abies alba) [34]. "

The phase "genome obesity threshold" is very odd (p. 4). It needs to be defined and explained, or else the sentence containing it should be deleted.

Typo: "Dnmt1-ty defense pe enzyme". I don't know how to correct this.

Typo: This should not be in all caps: "DECREASED DNA METHYLATION 1(DDM1)".

"gymnospermous genome" does not make sense (p. 5). I think they mean "gymnosperm genomes."

I don't understand this sentence: "Inevitably, the genomes become 'trapped' in mode as a "one way ticket to genomic obesity" [71,73]." Maybe the references explain it, but I don't have the time to go and read those. I don't know what "genomic obesity" is, and I don't know why the authors say genomes become "trapped" in anyway. They need to explain this clearly or else just delete the sentence.

This phrase is very odd: "poorer species richness in gymnosperms" (p. 6). I think they mean that gymnosperms don't have a large number of species compared to angiosperms, but the phrase doesn't really mean that. Needs to be re-written.

Top of p. 7, "Ks information" needs to be defined. I'm pretty sure I know what they mean, but "Ks information" does not serve as the way to describe the different rates of mutation in coding regions versus noncoding regions.
Top of p. 8, another odd phrase, "two old-age pulsed rises." I think "old-age" should simply be "ancient," but "old-age" is the wrong term to use for that. And I can only guess what a "pulsed rise" is, but really this must be re-written.
P. 10, bottom half: I don't believe this claim: "long genes tend to have higher expression levels in P. tabuliformis, which is similar to the pattern in P. glauca, Oryza sativa, and A. thaliana [26,105]." There have been studies that made this claim, but other studies have pointed out that there can be a strong bias in RNA-sequencing that makes it easy to over-count reads from long transcripts. It isn't clear what biological reason would explain this phenomenon, if it is real, so I am skeptical. The claim shouldn't be repeated here without a more thorough examination of the literature.
P. 10, middle, this sentence is ungrammatical, even though it is in quotes: "Why some genomes really big and others quite compact ? ". The following sentence uses "interrogated" incorrectly.
In the references, the journal G3 is shown as "G3 (Bethesda)". This is not correct. The journal is called "G3: Genes, Genomes, Genetics."

Methods

Are the methods appropriate to the aims of the study, are they well described, and are necessary controls included? Choose an item.

Conclusions

Are the conclusions adequately supported by the data shown? Choose an item.

Reporting Standards

Does the manuscript adhere to the journal's guidelines on minimum standards of reporting? Choose an item.

Choose an item.

Statistics

Are you able to assess all statistics in the manuscript, including the appropriateness of statistical tests used? Choose an item.

Quality of Written English

Please indicate the quality of language in the manuscript: Choose an item.

Declaration of Competing Interests

Please complete a declaration of competing interests, considering the following questions:

- Have you in the past five years received reimbursements, fees, funding, or salary from an organisation that may in any way gain or lose financially from the publication of this manuscript, either now or in the future?
- Do you hold any stocks or shares in an organisation that may in any way gain or lose financially from the publication of this manuscript, either now or in the future?
- Do you hold or are you currently applying for any patents relating to the content of the manuscript?
- Have you received reimbursements, fees, funding, or salary from an organization that holds or has applied for patents relating to the content of the manuscript?
- Do you have any other financial competing interests?
- Do you have any non-financial competing interests in relation to this paper?

If you can answer no to all of the above, write 'I declare that I have no competing interests' below. If your reply is yes to any, please give details below.

I declare that I have no competing interests

I agree to the open peer review policy of the journal. I understand that my name will be included on my report to the authors and, if the manuscript is accepted for publication, my named report including any attachments I upload will be posted on the website along with the authors' responses. I agree for my report to be made available under an Open Access Creative Commons CC-BY license (http://creativecommons.org/licenses/by/4.0/). I understand that any comments which I do not wish to be included in my named report can be included as confidential comments to the editors, which will not be published.

Choose an item.

To further support our reviewers, we have joined with Publons, where you can gain additional credit to further highlight your hard work (see: https://publons.com/journal/530/gigascience). On publication of this paper, your review will be automatically added to Publons, you can then choose whether or not to claim your Publons credit. I understand this statement.

Yes Choose an item.
