# Supplementary material for: Evolution of complex genome architecture in gymnosperms
Source: Gigascience. 2022 Aug 10;11:giac078. doi: 10.1093/gigascience/giac078 (PMC9364684; doi:10.1093/gigascience/giac078)
Supplement: giac078_Reviewer_1_Report_Revision_1 — Steven L. Salzberg, Ph.D. -- 6/21/2022 Reviewed [file giac078_reviewer_1_report_revision_1.pdf]

Reviewer Report

Title: Evolution of complex genome architecture in gymnosperms

Version: Revision 1 **Date:** 6/21/2022

Reviewer name: Steven L. Salzberg, Ph.D.

Reviewer Comments to Author:

The authors have done a very good job addressing most of my concerns, but there is still a bit more to do. I appreciate that they added information to Table 1 including a column linking to the actual genome data. However, that column is incomplete in most cases - the links are far too generic, going to a top-level database website (for example) such as plantgenie.org, which doesn't show me where the data is. All links should point to a directory that clearly contains the genome in question. Examples (I'm not going through the whole table, because that's for the authors to do):

1. Pinus taeda on row 4 points to https://treegenesdb.org/FTP/Genomes/. That directory has dozens of short abbreviations and it's not clear which one is P. taeda. I figured out that "Pita" is the right one, so Table 1 should link to https://treegenesdb.org/FTP/Genomes/Pita/.
2. Plantgenie.org is the top level page for that website. I looked for Picea abies (row 2 of Table 1), which I eventually found at https://plantgenie.org/FTP?dir=Data%2FConGenIE%2FPicea_abies%2Fv1.0%2FFASTA%2FGenomeAssemblies. However, that's a very peculiar link, and it's not likely to be stable. This points out a much better solution: all of these links should point to the specific NCBI/EMBL/DDBJ accession number, which is a stable, universal accession that should work for any published genome (and some unpublished ones). All journals require submission of genome data to one of those 3 databases, which form the International Nucleotide Sequence Database Collaboration, so the data should always be available at INSDC, with no restrictions. (Note that Nature just recently added the NGDC database in China as an acceptable repository.) So the authors should point to those links in Table 1.
For P. abies, the NCBI link is https://www.ncbi.nlm.nih.gov/nuccore/CBVK0000000000.1.
3. This also leads me to point out that it's not OK to just link to NCBI, as is done for Larix sibirica and a few others. A quick search finds this one at the following accession: https://www.ncbi.nlm.nih.gov/nuccore/NWUY0000000000.1. Note that the BioProject link at NCBI is just as good, so the authors could also use https://www.ncbi.nlm.nih.gov/bioproject/PRJNA393226. The BioProject has other useful information on the same page.

Note that it would be preferable if the authors could replace plantgenic.org, gigadb.org, and treegenesdb.org with the NCBI/EMBL accession numbers (and links), which is more reliably available over time. However it is okay if the just create more-specific links for those websites.

Methods

Are the methods appropriate to the aims of the study, are they well described, and are necessary controls included? Choose an item.

Conclusions

Are the conclusions adequately supported by the data shown? Choose an item.

Reporting Standards

Does the manuscript adhere to the journal's guidelines on minimum standards of reporting? Choose an item.

Choose an item.

Statistics

Are you able to assess all statistics in the manuscript, including the appropriateness of statistical tests used? Choose an item.

Quality of Written English

Please indicate the quality of language in the manuscript: Choose an item.

Declaration of Competing Interests

Please complete a declaration of competing interests, considering the following questions:

- Have you in the past five years received reimbursements, fees, funding, or salary from an organisation that may in any way gain or lose financially from the publication of this manuscript, either now or in the future?
- Do you hold any stocks or shares in an organisation that may in any way gain or lose financially from the publication of this manuscript, either now or in the future?
- Do you hold or are you currently applying for any patents relating to the content of the manuscript?
- Have you received reimbursements, fees, funding, or salary from an organization that holds or has applied for patents relating to the content of the manuscript?
- Do you have any other financial competing interests?
- Do you have any non-financial competing interests in relation to this paper?

If you can answer no to all of the above, write 'I declare that I have no competing interests' below. If your reply is yes to any, please give details below.

I declare that I have no competing interests

I agree to the open peer review policy of the journal. I understand that my name will be included on my report to the authors and, if the manuscript is accepted for publication, my named report including any attachments I upload will be posted on the website along with the authors' responses. I agree for my

report to be made available under an Open Access Creative Commons CC-BY license (http://creativecommons.org/licenses/by/4.0/). I understand that any comments which I do not wish to be included in my named report can be included as confidential comments to the editors, which will not be published.

Choose an item.

To further support our reviewers, we have joined with Publons, where you can gain additional credit to further highlight your hard work (see: https://publons.com/journal/530/gigascience). On publication of this paper, your review will be automatically added to Publons, you can then choose whether or not to claim your Publons credit. I understand this statement.

Yes Choose an item.
