# Supplementary material for: Evolution of complex genome architecture in gymnosperms
Source: Gigascience. 2022 Aug 10;11:giac078. doi: 10.1093/gigascience/giac078 (PMC9364684; doi:10.1093/gigascience/giac078)
Supplement: giac078_Reviewer_2_Report_Original_Submission — David Neale -- 6/3/2022 Reviewed [file giac078_reviewer_2_report_original_submission.pdf]

Reviewer Report

Title: Evolution of complex genome architecture in gymnosperms

Version: Original Submission **Date:** 6/3/2022

Reviewer name: David Neale

Reviewer Comments to Author:

I agreed to review this paper based on what was stated in the title and abstract. This suggested the paper would be a comprehensive review and comparative analysis of all elements of currently published gymnosperm genome sequences. In fact, the paper is detailed discussion of just three aspects of these genome sequences; (1) repetitive sequences, (2) whole genome duplications and (3) intron length. There is absolutely no discussion of the coding gene content of these genomes.

I suggest the authors and journal editors take one of two options. 1. Rename the title of this paper and rewrite the abstract and then have the paper reviewed by experts in 1-3 above. I am not such an expert. 2. Revise the paper so it includes all aspects of genome sequences, notably the gene space.

Also, the timeline of public release of conifer genome sequences given in this paper is totally wrong. The loblolly pine reference genome sequencing project was funded in 2010 and began in 2011. In early 2013, a draft assembly was released. The Norway spruce genome was published later that year, only half the genome actually sequenced and the assembly was of very poor quality.

Methods

Are the methods appropriate to the aims of the study, are they well described, and are necessary controls included? Choose an item.

Conclusions

Are the conclusions adequately supported by the data shown? Choose an item.

Reporting Standards

Does the manuscript adhere to the journal's guidelines on [minimum standards of reporting?](#) Choose an item.

Choose an item.

Statistics

Are you able to assess all statistics in the manuscript, including the appropriateness of statistical tests used? Choose an item.

Quality of Written English

Please indicate the quality of language in the manuscript: Choose an item.

Declaration of Competing Interests

Please complete a declaration of competing interests, considering the following questions:

- Have you in the past five years received reimbursements, fees, funding, or salary from an organisation that may in any way gain or lose financially from the publication of this manuscript, either now or in the future?
- Do you hold any stocks or shares in an organisation that may in any way gain or lose financially from the publication of this manuscript, either now or in the future?
- Do you hold or are you currently applying for any patents relating to the content of the manuscript?
- Have you received reimbursements, fees, funding, or salary from an organization that holds or has applied for patents relating to the content of the manuscript?
- Do you have any other financial competing interests?
- Do you have any non-financial competing interests in relation to this paper?

If you can answer no to all of the above, write 'I declare that I have no competing interests' below. If your reply is yes to any, please give details below.

I declare that I have no competing interests

I agree to the open peer review policy of the journal. I understand that my name will be included on my report to the authors and, if the manuscript is accepted for publication, my named report including any attachments I upload will be posted on the website along with the authors' responses. I agree for my report to be made available under an Open Access Creative Commons CC-BY license (http://creativecommons.org/licenses/by/4.0/). I understand that any comments which I do not wish to be included in my named report can be included as confidential comments to the editors, which will not be published.

Choose an item.

To further support our reviewers, we have joined with Publons, where you can gain additional credit to further highlight your hard work (see: https://publons.com/journal/530/gigascience). On publication of this paper, your review will be automatically added to Publons, you can then choose whether or not to claim your Publons credit. I understand this statement.

Yes Choose an item.
