# Supplementary material for: Evolution of complex genome architecture in gymnosperms
Source: Gigascience. 2022 Aug 10;11:giac078. doi: 10.1093/gigascience/giac078 (PMC9364684; doi:10.1093/gigascience/giac078)
Supplement: giac078_Authors_Response_To_Reviewer_Comments [file giac078_authors_response_to_reviewer_comments.pdf]

Author's Response To Reviewer Comments

Close

1.The title and the abstract have been substantially revised and the text has been polished again by another native speaker (Dr. Ruth from UK).
2.All web links and URLs in main text have been given a reference number and included in the reference list.
3.We have updated all the links in Table 1 to make the data be easily accessed by readers.

Close
